# Silole allylic anions instead of silanides†‡

**DOI:** 10.1039/d1dt02363b

**Published:** 2021-11-05

**Authors:** Alexander Pöcheim, Lena Albers, Thomas Müller, Judith Baumgartner, Christoph Marschner

**Affiliations:** Institut für Anorganische Chemie, Technische Universität Graz 8010 Graz Austria, European Union baumgartner@tugraz.at christoph.marschner@tugraz.at; Institut für Chemie, Carl von Ossietzky Universität Oldenburg 26111 Oldenburg Germany, European Union

## Abstract

Reaction of a 3,4-diphenylsilole with two neopentasilanyl groups attached to the 2- and 5-positions with one equivalent of KO^*t*^Bu did not result in the expected silanide formation but yielded a silole allylic anion instead. The initially formed silanide added to a neighboring phenyl group, which then transfers a proton to the 2-position of the silole ring.

## Abstract 2

The reactions of 3,4-diphenylsiloles with two neopentasilanyl groups in 2- and 5-positions with one equivalent of KO^*t*^Bu do not yield the expected silanides but silole allylic anions instead.

## Graphical abstract

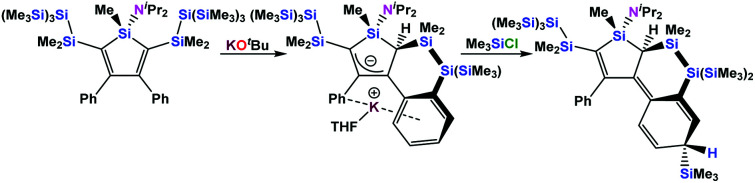

Over the last two decades siloles, that are 1-sila-2,4-cyclopentadienes,^1–5^ have been recognized as an unusually interesting class of compounds. Much of this interest is associated with their electron and hole transporting properties^6,7^ and the phenomenon of aggregation-induced-emission (AIE).^8–10^

Most of the intriguing properties of siloles are connected to their type of conjugation, namely cross-hyperconjugation or σ*–π*-conjugation.^11^ While the combination of silole cross-hyperconjugation with π-conjugated systems, like attached or annulated aryl groups, is well known, we were curious whether mixed conjugation of siloles would also be possible with σ-conjugated oligosilanyl units. This is indeed the case and we recently reported on the synthesis and conjugational properties of 2,5-di(oligosilanylated) 1,1-dimethyl-3,4-diphenylsilole **1a**, which constitutes a first example of the combination of cross-hyperconjugation and σ-conjugation in 2,5-oligosilanylated siloles.^12^

The current account deals with experiments to modify the oligosilanyl parts of siloles like **1a**. The possibility of converting oligosilanyl groups to silanides would reveal their potential to serve as building blocks for the construction of more complex molecular architectures. The presence of two neopentasilanyl units attached to the silole unit of compound **1a** suggests the possible preparation of oligosilanides *via* reactions with KO^*t*^Bu.^13–16^ Silanides are formidable nucleophilic building blocks for the extension or modification of oligosilanes.^13^ The two neopentasilanyl groups of **1a** are well separated, and based on our experience with similar compounds we assumed that formation of both mono- and dianionic compounds **K[2a]** and **K2[3a]** from compound **1a** should be possible employing either one or two equivalents of KO^*t*^Bu (Scheme 1).

**Scheme 1:**
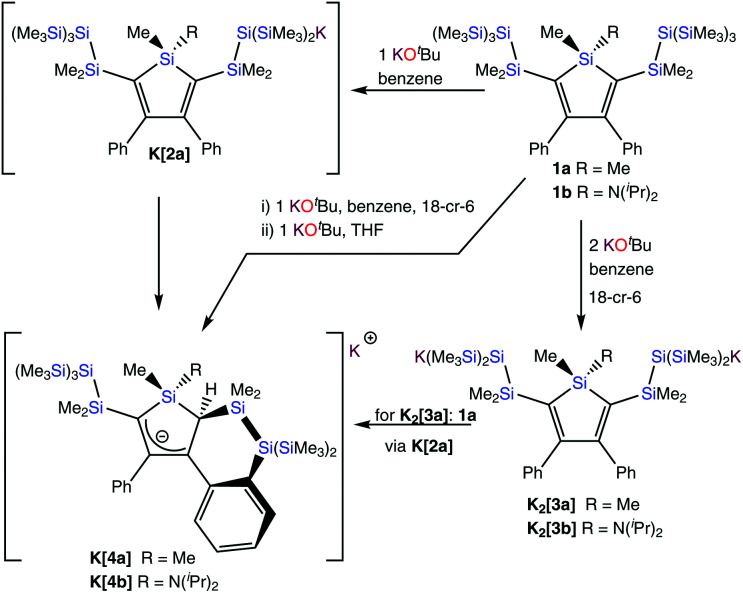
Reaction of the bis(neopentasilanyl) substituted siloles **1a** and **1b** with either two or one molar equivalents of KO^*t*^Bu to compounds **K2[3a]** or **K[4a]** and **K2[3b]** or **K[4b]**, respectively.

Reacting **1a** with two equivalents of KO^*t*^Bu in the presence of 18-crown-6 at ambient temperature in benzene, indeed provided the expected dianionic compound **K2[3a]** quantitatively within a few hours (Scheme 1). The dianionic character of **K2[3a]** is clearly reflected in the ^29^Si NMR spectrum. A strongly up-field shifted signal at *δ* = −183.6 ppm is typical for trisilylated silanides and also the resonance at *δ* = −4.5 ppm for trimethylsilyl groups attached to a silanide is an expected value. The silole ^29^Si resonance of **K2[3a]** at *δ* = 19.5 ppm is close to the respective value of **1a** (20.7 ppm) indicating no immediate electronic influence of the silanide on the silole system.

The solid state structure of **[K(18-crown-6)]2[3a]**, determined by single crystal XRD analysis (Fig. S1‡) due to mediocre data set and a strong disorder in one of the crown ether molecules only provides a conformational picture. It shows that the *syn*-conformation of the oligosilanyl units, observed for **1a** changes to an *anti*-conformation in **[K(18-crown-6)]2[3a]**. This is a common pattern for dianionic oligosilanes, where the negatively charged silanide units tend to depart from each other as far as possible for electrostatic reasons. The potassium ions, each embedded into a crown ether unit, coordinate to the silanides with parts of the crown ethers occupying the space above and below the silole ring.

When the reaction of **1a** was carried out with only one equivalent of KO^*t*^Bu and crown ether, the formation of a small amount of **[K(18-crown-6)]2[3a]** was observed together with a new product **K[4a]**, which does not show the expected NMR signature of the hypothetical monosilanide compound **K[2a]** (Scheme 1). Reacting **1a** with one equivalent of KO^*t*^Bu in THF allowed clean formation of the unexpected compound **K[4a]** (Scheme 1). The ^1^H NMR spectrum of the new compound shows nine different methyl signals with different intensities in the region between 0 and 1 ppm. In addition, a single proton was detected at *δ* = 1.43 ppm. The protons of the two phenyl groups were found to be magnetically non-equivalent. This picture was supported by the ^13^C NMR spectrum, which suggests that the symmetry in one of the phenyl groups is broken to give inequivalent *ortho*- and *meta*-carbon atoms and in addition, an unexpected signal at *δ* = 31.3 ppm was detected. The ^29^Si NMR spectrum of **K[4a]** features eight inequivalent silicon atoms with a signal at *δ* = 16.5 ppm, suggesting that the silole unit is largely intact and typical resonances for a silylated tris(trimethylsilyl)silyl group at *δ* = −9.7 and −136.2 ppm. A signal at *δ* = −90.0 ppm was assigned to a trisilylated silicon atom with an additional phenyl substituent. Two trimethylsilyl (−14.1 and −20.1 ppm) and one dimethylsilyl (−16.1 ppm) groups need to be attached to this silicon atom, which leaves one dimethylsilyl (−11.5 ppm) signal for the bridge between the tris(trimethylsilyl)silyl group and the silole core. Despite the deeply colored solution, the missing up-field resonance in the ^29^Si NMR spectrum clearly excludes the presence of a silanide.

Single crystal XRD structure analysis of **K[4a]** (Fig. S2‡) eventually shed light onto this somewhat puzzling data. Despite of being again of mediocre quality, the structure solution clearly shows that compound **K[4a]** is not a silanide but rather the allylic carbanion depicted in Scheme 1.

In the solid state, **K[4a]** exists as a cluster composed of four molecular units, which are connected to each other by additional coordinative interactions of the potassium ion to either phenyl groups, double bonds or trimethylsilyl groups of a neighboring molecule. The molecular unit of **K[4a]** features a bond between the silicon atom that initially was supposed to bear the negative charge and an *ortho*-carbon atom of the phenyl group in 3-position. The C-2 carbon atom of the silole is sp^3^ hybridized and attached to an additional proton, which likely is the previous *ortho*-hydrogen of the now silylated phenyl group.

The formation of **K[2a]** seems to be the first step in the formation of **K[4a]**, which is supported by the fact that the reaction of the disilanide **K2[3a]** with an equimolar amount of **1a** also gives **K[4a]** (Scheme 1). This most likely occurs *via* silyl group exchange between silanide **K2[3a]** and silane **1a** to **K[2a]**, which is a very common process in the reactions of trimethylsilylated silanes with KO^*t*^Bu.^17^ Use of **K2[3a]** introduces two equivalents of 18-crown-6. The product formed in the reaction of **K2[3a]** with **1a**, therefore features the same or a very similar NMR signature as the one formed from **1a** with KO^*t*^Bu in THF, but the interaction of the potassium ion with the allylic anion is likely diminished. All attempts to obtain crystals from the reaction of **K2[3a]** with **1a** were unsuccessful, since a strong tendency of the product to separate as an oily residue was observed.

Transmetallation with magnesium bromide is used to moderate the reactivity of silanides.^18,19^ Although compound **K[4a]** is not a silanide, it was treated anyway with MgBr_2_·Et_2_O in THF to give **BrMg[4a]** (Fig. 1). In contrast to the structure of **K[4a]**, where the potassium ion is coordinated to phenyl groups of at least two allylic anions, we observe separated ion pairs for **[BrMg(THF)5][4a]**, where the allylic anion unit does not undergo interactions with the cationic [BrMg(THF)_5_]^+^ unit.

**Fig. 1:**
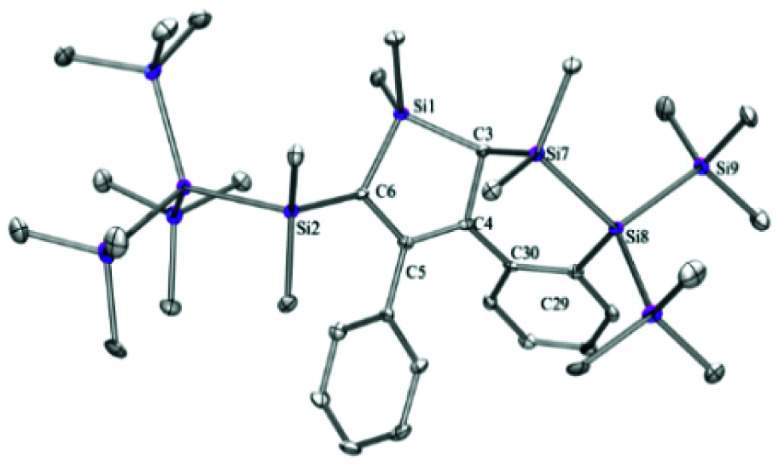
Molecular structure of the anionic part of **[BrMg(THF)5][4a]**, with the [BrMg(THF)_5_]^+^ unit, a THF molecule and hydrogen atoms omitted for clarity (thermal ellipsoid plot drawn at the 30% probability level.

The solid state structures of **K[4a]** and **[BrMg(THF)5][4a]**, as well as the calculated **[4a]−** (*vide infra*) suggest that there is a single bond between carbon atoms C-2 and C-3 and that the bond between C-4 and C-5 is no clear-cut double bond any more and also the bonds between C-3 and C-4 and the one between C-3 and the attached phenyl *ipso*-carbon atom are somewhere in between single and double bonds. Especially in the structure of **K[4a]**, bonds between *ortho*- and *meta*-carbon atoms of the newly silylated phenyl group at C-3 are slightly shorter than the other ones, suggesting some 1,4-diene character. Overall, the structure of **[4a]−** fulfills the structural requirements for a carbanion described with the resonance structure **A** with contributions of **B** and **C** in Chart 1.

**Chart 1:**
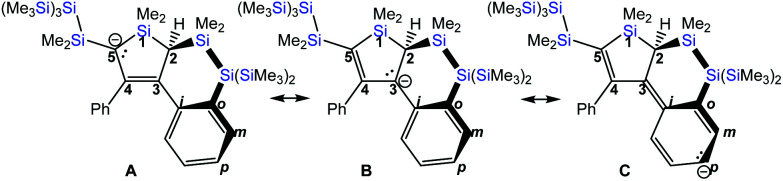
Resonance contributors to **[4a]−** as derived from **K[4a]**, **BrMg[4a]** and the calculated structure **[4a]−**.

To probe whether the allylic anion formation is specific to substrate **1a**, the amino substituted silole **1b** was subjected to the same conditions. Reaction with two equivalents of KO^*t*^Bu in the presence of crown ether in benzene gave the expected dianionic compound **[K(18-crown-6)]2[3b]** (Scheme 1). Also the reaction with one equivalent of KO^*t*^Bu in THF proceeded as observed previously for **1a** (Scheme 1) to give compound **K[4b]** as the sole product. NMR spectra of **[K(THF)][4b]** resemble that of **K[4a]** with a methyl group missing and additional triisopropyl and THF signals.

The fact that we do not observe an isomeric mixture indicates that the allylic carbanion formation occurs selectively in a way that distinguishes the two different sides of the silole ring. Again single crystal XRD analysis of **[K(THF)][4b]** provided the structural details (Fig. 2). The compound crystallized in a monomeric fashion with one THF molecule coordinated to the potassium ion, which is interacting with the two phenyl groups. The shifted proton is located on the same side as the diisopropylamino group so that the SiMe_2_ unit is pointing to the other side.

**Fig. 2:**
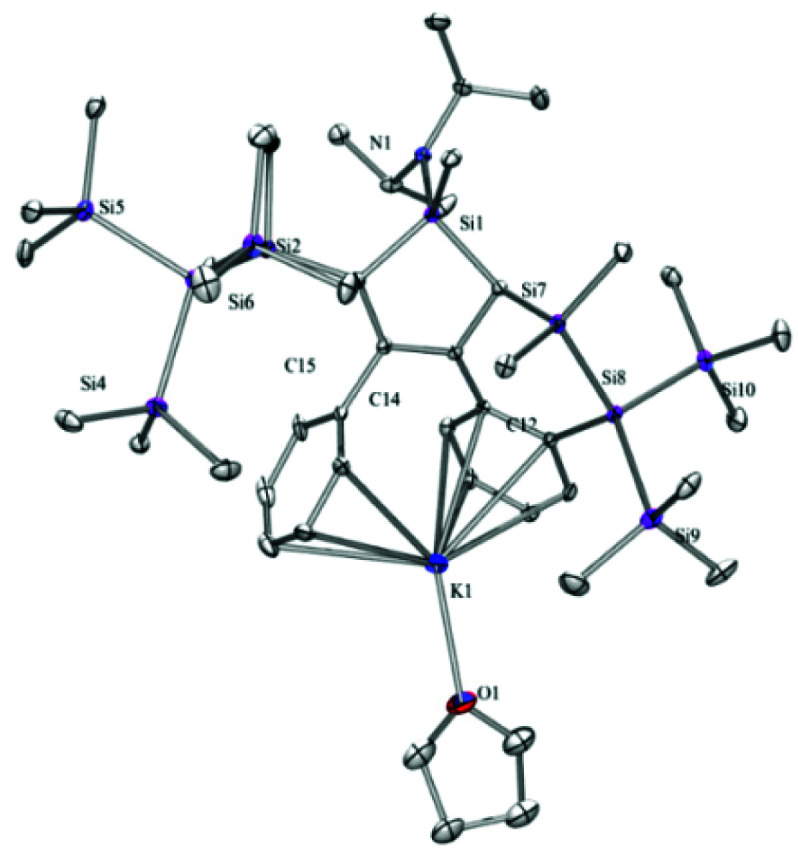
Molecular structure of **[K(THF)][4b]** (thermal ellipsoid plot drawn at the 30% probability level). All hydrogen atoms are omitted for clarity.

Reaction of compound **[K(THF)][4b]** with chlorotrimethylsilane gave compound **5b** as a single isomer (Scheme 2). Initially we expected to find the trimethylsilyl group of **5b** to be attached to either position 3- or 5- of the silole. Surprisingly, single crystal XRD analysis of **5b** (Fig. 3), showed it to be bound to the former *para*-carbon atom of the silylated phenyl group. This finding clearly suggests that resonance structure **C** in Chart 1 seems to be a significant contributor. The stereoselective attack onto the ^i^Pr_2_N side of the silole, might be explained by the potassium coordination, shielding the opposite side as shown nicely in the structure of **[K(THF)][4b]** (Fig. 2).

**Scheme 2:**
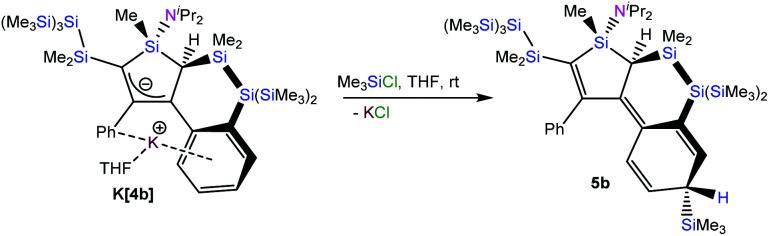
Reaction of compound **K[4b]** with chlorotrimethylsilane to compound **5b**.

**Fig. 3:**
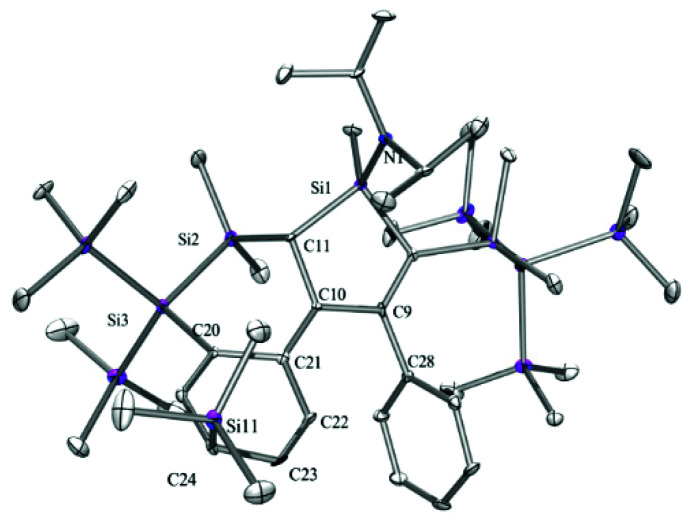
Molecular structure of **5b** (thermal ellipsoid plot drawn at the 30% probability level). All hydrogen atoms are omitted for clarity.

Reaction of allylic anion **K[4a]** with chlorotrimethylsilane proceeds less cleanly. Although it is possible to confirm the formation of a product **5a** with a very similar NMR signature to **5b**, the course of the reaction was less clean, suggesting diminished selectivity in the addition reaction. The reasons for this are not immediately obvious. The formation of **5b** was unexpected because based on literature evidence^20–23^ we assumed addition to the C-5 position. This was likely suppressed by the bulky neopentasilanyl and N(^i^Pr)_2_ substituents shielding both sides of the ring. Attack at the *para*-position of the phenyl is therefore kinetically favored.

For the formation of allylic anion [**4a**]^−^ we suggest fast initial formation of mono-silyl anion [**2a**]^−^ by reaction of **1a** with KO^*t*^Bu. Reaction with a second equivalent of KO^*t*^Bu to form the bis-silyl dianion [**3a**]^2−^ is faster than intramolecular nucleophilic addition of the silanide to the neighboring phenyl group with formation of an anionic Meisenheimer-type complex [**6**]^−^ (Schemes 3 and S1, Fig. S24 and S25‡). In the absence of a second equivalent of KO^*t*^Bu, the intramolecular reaction prevails and in a subsequent 1,4-proton shift the allylic anion [**4a**]^−^ is formed. DFT calculations show that the overall reaction of the mono-silyl anion [**2a**]^−^ to the allylic anion [**4a**]^−^ is strongly exothermic (Δ*E* = −101 kJ mol^−1^) and exergonic (Δ*G*(298) = −82 kJ mol^−1^) (Schemes 3 and S1‡). In agreement with the slow formation of [**4a**]^−^, the initiating nucleophilic addition step to [**6**]^−^ is endothermic (Δ*E* = 69 kJ mol^−1^, Δ*G*(298) = 79 kJ mol^−1^) (Scheme S1‡). The calculated shape of the HOMO of anion [**6**]^−^ suggests already the following 1,4-proton shift, as it has large contribution of atomic orbitals of carbon atom C-2 (Fig. S25‡). A complete reaction coordinate was obtained for the model anion [**2M**]^−^ to the allylic anion model [**4M**]^−^ (Fig. S24‡). Also the model reaction is strongly exothermic and formation of the intermediate [**6M**]^−^ is endothermic, although the smaller degree of substitution at the anionic silicon atom decreases the energy difference to the starting material. The rate determining step is the 1,4-proton shift from the *ortho-*position of the former phenyl substituent to the 2-position of the silole ring. The involved barriers (Δ*E* = 93 kJ mol^−1^ for the rate determining step) are in agreement with a reaction that proceeds slowly at room temperature (Fig. S24‡). The calculated surface diagram of the HOMO of allylic anion [**4a**]^−^ reveals its location mainly at the ring carbon atoms C-3 and C-5 with small delocalization tails into the terminal phenyl ring (Fig. S25‡).

**Scheme 3:**
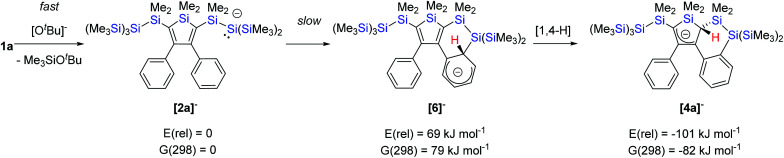
Suggested reaction mechanism for the formation of allylic anion **[4a]−** from the silyl anion **[2a]−**. Relative energies, *E*(rel), and Gibbs enthalpies at 298 K *G*(298) are calculated at the M06-2X/def2-TZVP level.

According to the results of our calculations for the isomeric trimethylsilyl derivatives of allylic anion [**4a**]^−^, the exclusive formation of the trimethylsilyl-trapping product **5b** from allylic anion [**4b**]^−^ is of kinetic origin as there is no clear thermodynamic preference for the different regioisomers of Me_3_Si[**4a**] (see Table S4‡). This view is supported by the observed diminished selectivity of the reaction of [**4a**]^−^ with chlorotrimethylsilane.

Siloles are prone to the attack of strong nucleophiles such as silanides or hydrides at the 2-position leading to the formation of silole allylic anions.^20–23^ In an attempt to convert a neopentasilanyl group of a 2,5-dioligosilanyl-3,4-diphenylsilole into a silanide, we observed that the formed compound undergoes an intramolecular nucleophilic attack at the *ortho*-carbon atom of the phenyl group in 3-position. A transient anionic Meisenheimer-type complex undergoes a 1,4-proton shift to the 2-position of the silole whereupon an annulated disilacyclohexane ring and an allylic anion are formed. This occurs only for mono-silanide formation but is suppressed when both neopentasilanyl groups are converted to silanides, suggesting that the observed intramolecular sequence of nucleophilic attack and 1,4-proton shift to the 2-position is thermodynamic disfavored compared to a competing second silanides formation.

## Conflicts of interest

There are no conflicts to declare.

## Supplementary Material

DT-050-D1DT02363B-s001

DT-050-D1DT02363B-s002
