# Supplementary material for: Silole allylic anions instead of silanides
Source: Dalton Trans. 2021 Nov 5;50(46):16945–9. doi: 10.1039/d1dt02363b (PMC8631112; doi:10.1039/d1dt02363b)
Supplement: DT-050-D1DT02363B-s001 [file DT-050-D1DT02363B-s001.pdf]

Electronic Supplementary Material (ESI) for Dalton Transactions.
This journal is © The Royal Society of Chemistry 2021

Supporting Information

Silole Allylic Anions Instead of Silanides

Alexander Pöcheim,[a] Lena Albers,[b] Thomas Müller[b], Judith Baumgartner,*[a] and Christoph Marschner*[a]

[a]Institut für Anorganische Chemie, Technische Universität Graz, 8010 Graz, Republic of Austria, European Union

E-mail: baumgartner@tugraz.at; christoph.marschner@tugraz.at

[b]Institut für Chemie, Carl von Ossietzky Universität Oldenburg

26111 Oldenburg, Federal Republic of Germany, European Union

Table of Contents

1. Experimental details........ S1
2. Crystallographic data and ORTEP Plots........ S6
3. NMR Spectra........ S12
   1b........ S12
   K_2[3a]........ S15
   K_2[3b]........ S18
   K[4a]........ S21
   K[4b]........ S24
   5b........ S27
4. Computational Details........ S30
5. References........ S34

1. Experimental Details

General Remarks. All reactions involving air-sensitive compounds were carried out under an atmosphere of dry nitrogen or argon using either Schlenk techniques or a glove box. All solvents were dried using column based solvent purification system.[S1] All used chemicals were obtained from different suppliers and used without further purification. Computational details are given provided in the respective section 4 below (absolute energies of compounds shown in Schemes 3 and S1, Figures S24 and S25 and Tables S2-S4). Optimized structures are given in separate xyz-files.

Dichloro(diisopropylamino)methylsilane,[S2] tetrakis(trimethylsilyl)silane,[S3] (diisopropyl-amino)methyldi(phenylethynyl)silane,[S4] and 1,1-dimethyl-2,5-bis[tris(trimethylsilyl)-silyldimethylsilyl]-3,4-di-phenyl¬silole (**1a**)[S5] have been prepared following published procedures.

^{1}H (300 MHz), ^{13}C (75.4 MHz), and ^{29}Si (59.3 MHz) NMR spectra were recorded on a Varian INOVA 300 spectrometer. Spectra are referenced to tetramethylsilane (TMS) for ^{1}H, ^{13}C, and ^{29}Si. If not noted otherwise all samples were measured in C_6D_6. To compensate for the low isotopic abundance of ^{29}Si the INEPT[S6,S7] or DEPT[S8] pulse sequences were used for the amplification of the signal. For cases where the silole Si signals could not be detected unambiguously the Varian s2pul sequence (inversed gated) was used. Elementary analyses were carried out using a Heraeus VARIO ELEMENTAR instrument. For metalated compounds no elemental analyses were attempted, as these are very sensitive and typically no meaningful can be obtained.

X-Ray Structure Determination. For X-ray structure analyses the crystals are mounted onto the tip of glass fibers, and data collection was performed with a BRUKER-AXS SMART APEX CCD diffractometer using graphite-monochromated Mo Kα radiation (0.71073 Å). The data were reduced to F^2_o and corrected for absorption effects with SAINT[S9] and SADABS,[S10,S11] respectively. The structures were solved by direct methods and using Olex2[S12] refined by full-matrix least-squares method (SHELXL).[S13] If not noted otherwise all non-hydrogen atoms were refined with anisotropic displacement parameters. All hydrogen atoms were located in calculated positions to correspond to standard bond lengths and angles. All diagrams are drawn with 30% probability thermal ellipsoids and all hydrogen atoms were omitted for clarity. Crystallographic data (excluding structure factors) for the structures of compounds **1b**, **[K(18-crown-6)]$_2$[3a]**, **K[4a]**, **[BrMg(THF)$_5$][4a]**, **[K(THF)][4b]**, and **5b** reported in this paper are deposited with the Cambridge Crystallographic Data Center as supplementary publication no. CCDC-

2075659 (**1b**), 2075663 (**[K(18-crown-6)]$_2$[3a]**), 2075664 (**K[4a]**), 2075661 **[BrMg(THF)$_5$][4a]**), 2075660 (**[K(THF)][4b]**), and 2075662 (**5b**). Copies of data can be obtained free of charge at: http://www.ccdc.cam.ac.uk/products/csd/request/. Figures of solid state molecular structures were generated using Ortep-3 as implemented in WINGX[S14] and rendered using POV-Ray 3.6.[S15]

1-(Diisopropylamino)-1-methyl-2,5-bis(chlorodimethylsilyl)-3,4-diphenylsilole.

To a solution of naphthalene (4.95 g, 4.8 equiv., 38.6 mmol) in THF (70 mL) small pieces of lithium ribbon (251 mg, 4.5 equiv., 36.2 mmol) were added. The malachite green solution was stirred for 5 h. Afterwards the solution was cooled to −80 °C and diisopropylaminomethyldi(phenylethynyl)silane (2.81 g, 1.0 equiv., 8.13 mmol) dissolved in THF (13 mL) was added dropwise. The solution was stirred at −85 °C for 65 min and a dark brown color was observed. Then dichlorodimethylsilane (6.63 g, 6.3 equiv., 51.4 mmol, 6.2 mL) was added very fast and the solution was allowed to warm up to rt after 10 min. The now dark brownish reaction mixture was stirred for 14 h. The volatiles were removed under reduced pressure. The residue was subjected to sublimation at 80 °C and 1.0 mbar to remove the majority of naphthalene (4.12 g, 83%). The remaining solid was extracted with pentane, centrifuged and filtered. The yellow solution was kept at −62 °C for crystallization. The product was obtained as yellow crystals (3.06 g, 71%). decomp.: 129-131 °C. NMR (δ in ppm): ^{1}H: 6.91 – 6.74 (m, 10H), 3.49 (hept, J = 6.8 Hz, 2H), 1.33 (d, J = 6.7 Hz, 12H), 0.88 (s, 3H), 0.32 (s, 6H), 0.18 (s, 6H). ^{13}C: 168.8, 143.6, 141.6, 128.9, 127.7, 127.2, 46.8, 25.1, 5.2, 4.9, −1.8. ^{29}Si: 16.9 (s, $SiMe_2$), 9.9 (s, SiMe). Anal. calcd. for $C_{27}H_{39}Cl_2NSi_3$ (532.77): C 60.87, H 7.38, N 2.63. Found: C 60.55, H 7.22, N, 2.45. UV-Vis: $\lambda_{max,1}$: 272.0 nm, ε = 5.65 × 10^3 M^{-1} cm^{-1}; $\lambda_{shoulder,1}$: 278.0 nm, ε = 5.55 × 10^3 M^{-1} cm^{-1}; $\lambda_{shoulder,2}$: 291.0 nm, ε = 5.14 × 10^3 M^{-1} cm^{-1}; $\lambda_{shoulder,3}$: 302.5 nm, ε = 4.77 × 10^3 M^{-1} cm^{-1}.

1-(Diisopropylamino)-1-methyl-2,5-bis[dimethyl{tris(trimethylsilyl)silyl}silyl)]-3,4-diphenylsilole (1b).

A solution of tetrakis(trimethylsilyl)silane (331 mg, 1.03 mmol, 2.06 equiv.) and KO^{t}Bu (118 mg, 1.05 mmol, 2.10 equiv.) in THF (2 mL) was kept at rt for 3 h, until NMR spectroscopy showed full conversion toward tris(trimethylsilyl)silyl potassium. Volatiles were removed under reduced pressure and toluene (2 mL) was added to give an orange solution. The dissolved silanide was added dropwise within 2 min to a solution of **2b** (267 mg, 0.50 mmol, 1.00 equiv.) in toluene (2 mL) and was stirred at rt for 17 h, after which full conversion was observed with NMR analysis. Solvents were removed to give a bright

violet residue. The residue was extracted with pentane (3 × 3 mL), centrifuged and filtered. Yellow crystals (420 mg, 87%) of **1b** were obtained from a pentane solution at −62 °C. mp.: 245-247 °C NMR (δ in ppm): ^{1}H: 7.14 - 6.64 (m, 10H), 3.69 (hept, *J* = 7.1 Hz, 2H), 1.37 (d, *J* = 6.9 Hz, 12H), 0.91 (s, 3H), 0.81 (s, 6H), 0.34 (s, 54H), 0.17 (s, 6H). ^{13}C: 170.0, 143.2, 142.8, 128.1, 127.7, 126.6, 48.0, 26.1, 6.8, 6.4, 4.4, −0.2. ^{29}Si: 13.8 (s, SiMe), −9.4 (s, $SiMe_3$), −19.4 (s, $SiMe_2$), −126.7 (s, Si_q). Anal. calcd. for $C_{45}H_{93}NSi_{11}$ (957.18): C 56.47, H 9.79, N 1.46. Found: C 56.30, H 9.78, N 1.41. UV-Vis: λ_{max}: 284 nm, ε = 7.69 × 10^{3} M^{-1} cm^{-1}; λ_{max}: 338 nm, ε = 6.59 × 10^{3} M^{-1} cm$^{-1;}$ $\lambda_{shoulder,1}$: 383.0 nm, ε = 1.6 × 10^{3} M^{-1} cm^{-1}.

1,1-Dimethyl-2,5-bis[dimethyl{potassio-bis(trimethylsilyl)silyl}silyl]-3,4-diphenylsilole ([K(18-crown-6)]$_2$[3a]).

1a (130 mg, 0.132 mmol), KO^{t}Bu (31 mg, 0.276 mmol), and 18-cr-6 (70 mg, 0.265 mmol) were dissolved in benzene (4 mL) and stirred at rt for 3 d. Immediately, the reaction mixture turned bright violet. After full conversion toward **K_2[3a]** was observed with NMR analysis, the solvent was removed under reduced pressure and the residue was dissolved in benzene (1 mL) and filtered. Violet crystals of **[K(18-crown-6)]$_2$[3a]** (160 mg, 91%) were obtained by slow addition of pentane. NMR (δ in ppm): ^{1}H: 7.54 – 7.44 (m, 2H), 7.22 – 7.03 (m, 8H), 3.26 (s, 48H), 0.99 (s, 6H), 0.75 (s, 36H), 0.50 (s, 12H). ^{13}C: 161.7, 155.6, 145.9, 130.9, 127.0, 125.2, 70.0, 9.1, 7.7, 1.8. ^{29}Si: 19.5 (s, silole-$SiMe_2$), −4.5 (s, $SiMe_3$), −12.0 (s, $SiMe_2$), −183.6 (s, Si_q).

Allylic anion resulting from the reaction of 1a with KO^{t}Bu (K[4a]).

With THF: Silole **1a** (100 mg, 0.115 mmol, 1.00 equiv.) and KO^{t}Bu (13 mg, 0.116 mmol, 1.01 equiv.) were dissolved in THF (1 mL) and were left at rt for 16 h. Immediately, the reaction mixture turned deep purple. The solvents were removed under reduced pressure and the bright violet residue was extracted with pentane (6 mL) and filtered. Deep purple crystals of **K[4a]** (75 mg, 72%) were obtained from this solution at rt. NMR (δ in ppm): ^{1}H (C_6D_6): 7.92 (bs, 1H), 7.31 (d, *J* = 5.7 Hz, 4H), 6.90 (m, 1

H), 6.59 (d, *J* = 8.1 Hz, 1H), 6.24 (t, *J* = 6.5 Hz, 1H), 6.17 (t, *J* = 6.5 Hz, 1H), 1.43 (s, 1H), 1.02 (s, 3H), 1.00 (s, 3H), 0.83 (s, 3H), 0.77 (s, 3H), 0.66 (s, 27H), 0.53 (s, 3H), 0.49 (s, 9H), 0.47 (s, 9H), 0.04 (s, 3H). ^{13}C (d^{8}-THF): 162.5, 158.2, 151.6, 138.4, 133.0 (2×), 132.7, 128.8, 126.9, 125.5, 124.0, 115.8, 109.9, 31.3, 11.9, 8.0, 6.6, 5.0, 2.8, 2.1, 1.9, 1.7, −0.7. ^{29}Si (C_6D_6): 16.5 (s, $SiMe_2$), −9.7 (s, Si(<u>Si</u>Me_3)$_3$), −11.5 (s, $SiMe_2$), −14.1 (s, $SiMe_3$), −16.2 (s, <u>Si</u>Me_2Si($SiMe_3$)$_3$), −20.1 (s, $SiMe_3$), −90.0 (s, <u>Si</u>($SiMe_3$)$_2$), −136.2 (s, <u>Si</u>($SiMe_3$)$_3$).

With 18-cr-6: Compound **1a** (99 mg, 0.113 mmol), KO^{t}Bu (13 mg, 0.116 mmol) and 18-cr-6 (33 mg, 0.125 mmol) were dissolved in benzene (1 mL) and left at rt. Immediately, the reaction mixture turned bright violet. After 2 d, NMR analysis showed the formation of **[K(18-crown-6)][4a]** and **[K(18-crown-6)]$_2$[3a]** along with unreacted **1a**. The solvents were removed under reduced pressure and the bright violet residue was extracted with pentane (3 × 1 mL) and filtered. Bright purple crystals of **[K(18-crown-6)][4a]** (64 mg, 51%) were obtained from the pentane solution at rt. NMR spectra are essentially identical with those from the reaction in THF (**K[4a]**) with an additional signal for the crown ether.

K[4a] formation from the combination of 1a and [K(18-crown-6)]$_2$[3a].

To a solution of **[K(18-crown-6)]$_2$[3a]** (40 mg, 0.030 mmol) in benzene (0.5 mL) was added a solution of **1a** (58 mg, 0.066 mmol) in benzene (0.5 mL). The purple solution was left for 3 weeks at rt. The reaction progress was monitored with NMR analysis and revealed a steady decline of **1a** and **[K(18-crown-6)]$_2$[3a]** along with the formation of **[K(18-crown-6)][4a]**.

1-(Diisopropylamino)-1-methyl-2,5-bis[dimethyl{potassio-bis(trimethylsilyl)silyl}silyl]-3,4-diphenylsilole ([K(18-crown-6)]$_2$[3b]).

A solution of **1b** (101 mg, 0.106 mmol, 1.00 equiv.), KO^{t}Bu (24 mg, 0.214 mmol, 2.03 equiv.) and 18-cr-6 (59 mg, 0.223 mmol, 2.11 equiv.) in benzene (2 mL) was allowed to react at rt for 20 h. Immediately, the reaction mixture turned bright red. Volatiles were removed under reduced pressure and pentane (1 mL) was added to remove non-polar side products. The colorless pentane solution was decanted and discarded. The remaining solid was dried in vacuo to give **K$_2$[3b]** (150 mg, >99%) as deep red, metallic looking foam. NMR (δ in ppm, d$_8$-THF): ^{1}H: 6.60-6.45 (m, 10H), 3.75 (hept, J = 6.9 Hz, 2H), 3.48 (s, 48H), 1.19 (d, J = 6.7 Hz, 12H), 0.85 (s, 3H), 0.15 (s, 6H), -0.13 (s, 36H), −0.62 (s, 6H). ^{13}C: 164.0, 151.6, 147.0, 129.3 (2 signals), 124.2, 71.3, 48.0, 26.8, 26.4, 9.4, f8.9, 8.6, 7.8, 3.6. ^{29}Si: 15.7 (s, silole-SiMe$_2$), −5.1 (s, SiMe$_3$), −12.0 (s, SiMe$_2$), −183.9 (s, Si$_q$).

Allylic anion resulting from the reaction of 1b with KO^{t}Bu ([K(THF)][4b]).

Compound **1b** (100 mg, 0.105 mmol) and KO^{t}Bu (24 mg, 0.214 mmol) were dissolved in THF (1 mL). Immediately, the solution turned orange and then deep brown. After 15 h, NMR analysis of the now red solution showed the exclusive formation of silyl ether and **K[4b]**. The solvent was removed under reduced pressure to give a violet residue, which was extractable with pentane. From this solution several batches of violet crystals of

[K(THF)][4b] (95 mg, 92%) were obtained at rt. NMR (δ in ppm): ^{1}H: 7.79 (bs, 1H), 7.28 (bs, 1H), 6.94 (d, *J* = 6.9 Hz, 1H), 6.87 (bs, 1H), 6.66 (t, *J* = 7.1 Hz, 1H), 6.58 (bs, 1H), 6.30 (d, *J* = 7.9 Hz, 1H), 6.16 (t, *J* = 6.8 Hz, 1H), 5.84 (t, *J* = 6.8 Hz, 1H), 3.85 (hept, *J* = 6.9 Hz, 2H), 3.42 (t, 6.6 Hz, 3H), 1.64 (s, 1H), 1.39 (d, *J* = 6.6 Hz, 6H), 1.37, 1.27 (d, *J* = 6.7 Hz, 6H), 1.05 (s, 3H), 0.92 (s, 3H), 0.74 (s, 3H), 0.67 (s, 6H), 0.44 (s, 27H), 0.36 (s, 9H), 0.14 (s, 9H). ^{13}C: 164.5, 156.2, 152.2, 138.9, 135.3 (bs), 132.4, 131.2 (bs), 129.6, 128.6, 128.2, 127.9, 126.2, 126.0, 125.0, 113.6, 113.1, 67.8, 45.2, 32.9, 25.7, 25.1, 24.9, 22.8, 14.3, 12.1, 8.4, 7.1, 4.7, 4.3, 3.0, 1.7, 1.4, −0.4. ^{29}Si: 14.0 (s, $SiMeN^iPr_2$), −10.0 (s, Si(<u>Si</u>Me$_3$)$_3$), −12.3 (s, $SiMe_3$), −12.7 (s, $SiMe_3$), −18.1 (s, <u>Si</u>Me$_2$Si(SiMe$_3$)$_3$), −23.3 (s, $SiMe_2$), −87.9 (s, <u>Si</u>(SiMe$_3$)$_2$), −130.5 (s, <u>Si</u>(SiMe$_3$)$_3$).

***rac*-(3R,3aS,7R)-*N,N*-Diisopropyl-3,4,4-trimethyl-2-dimethyl{tris(trimethylsilyl)silyl}silyl-1-phenyl-5,5,7-tris(trimethylsilyl)-3a,4,5,7-tetrahydro-3H-benzo[c]silolo[3,2-e][1,2]disilin-3-amine (5b).**

To a THF solution (1 mL) of allylic anion **[K(THF)][4b]** (35 mg, 0.0312 mmol) was added dropwise an excess of trimethylsilylchloride (300 mg, 2.76 mmol). The reaction mixture was left at rt for 20 h. The color changed from red to orange. After full conversion was observed with NMR analysis the volatiles were removed under reduced pressure. The residue was dissolved in pentane (3 × 1 mL) and filtered. Orange crystals of **5b** (30 mg, 91%) were obtained at rt. Mp: 203-205 °C (decomp., phase transition at 174-176 °C). Anal. calcd. for $C_{45}H_{93}NSi_{11}$ (957.18): C 56.47, H 9.79, N 1.46. Found: C 56.12, H 9.91, N, 1.37. NMR (δ in ppm): ^{1}H: 7.43 (d, *J* = 7.3 Hz, 2H), 7.20 (t, *J* = 7.3 Hz, 2H), 7.10 (t, *J* = 7.3 Hz, 1H), 6.41 (dd, *J* = 5.8, 1.5 Hz, 1H), 5.34 (d, *J* = 10.3 Hz, 1H), 5.30 (dd, *J* = 4.1, 1.4 Hz, 1H), 3.40 (hept, *J* = 6.8 Hz, 2H), 2.54 (t, *J* = 4.8 Hz, 1H), 1.27 (s, 1H), 1.23 (d, *J* = 4.3 Hz, 6H), 1.21 (d, *J* = 4.2 Hz, 6H), 0.91 (s, 3H), 0.90 (s, 3H), 0.64 (s, 3H), 0.57 (s, 3H), 0.55 (s, 3H), 0.39 (s, 9H), 0.30 (s, 27H), 0.27 (s, 9H), −0.06 (s, 9H). ^{13}C: 146.8, 145.8, 145.1, 142.5, 137.2, 132.2, 130.1, 128.8, 127.7, 127.6, 125.8, 125.7, 46.4, 40.5, 27.7, 25.1, 24.9, 9.8, 7.4, 5.9, 4.4, 2.3, 1.5, 0.9, −0.6, −2.6. ^{29}Si: 14.8 (s, $SiMeN^iPr_2$), −3.4 (s, $CHSiMe_3$), −9.4 (s, Si(<u>Si</u>Me$_3$)$_3$), −9.5 (s, $SiMe_3$), −11.7 (s, $SiMe_3$), −12.4 (s, $SiMe_2$), −20.9 (s, <u>Si</u>Me$_2$Si(SiMe$_3$)$_3$), −84.8 (s, <u>Si</u>(SiMe$_3$)$_2$), −124.6 (s, <u>Si</u>(SiMe$_3$)$_3$).

2. Crystallographic Information

Table S1. Crystallographic data for compounds **1b**, **[K(18-crown-6)]$_2$[3a], K[4a]**, **[BrMg(THF)$_5$][4a]**, **[K(THF)][4b]**, and **5b**.

	**1b**	**[K(18-crown-6)]$_2$[3a]**	**K[4a]**	**[BrMg(THF)$_5$][4a]**	**[K(THF)][4b]**	**5b**
Empirical formula	$C_{45}H_{93}NSi_{11}$	$C_{58}H_{112}K_2O_{12}Si_9$	$C_{74}H_{146}K_2Si_{20}$	$C_{61}H_{119}BrMgO_6Si_{10}$	$C_{46}H_{92}KNOSi_{10}$	$C_{45}H_{93}NSi_{11}$
M_w	957.19	1332.48	1675.91	1333.68	995.21	957.19
Temperature [K]	100(2)	100(2)	150(2)	150(2)	100(2)	100(2)
Size [mm]	0.28×0.18×0.10	0.32×0.30×0.14	0.28×0.22×0.13	0.22×0.18×0.10	0.46×0.22×0.12	0.44×0.17×0.11
Crystal system	monoclinic	Triclinic	triclinic	Triclinic	monoclinic	monoclinic
Space group	P2(1)/c	P-1	P-1	P-1	P2(1)/n	C2/c
a [Å]	22.157(4)	11.090(4)	16.82(3)	16.567(3)	17.893(4)	37.205(10)
b [Å]	20.831(4)	11.339(5)	17.503(3)	16.634(3)	13.437(3) A	13.523(3)
c [Å]	14.438(3)	34.641(2)	18.760(3)	17.495(3)	25.350(5)	25.072(6)
α [°]	90	88.349(6)	110.608(3)	101.384(4)	90	90
β [°]	99.511(3)	84.449(6)	93.224(3)	98.071(4)	91.669(4)	96.287(8)
γ [°]	90	61.697(5)	101.578(3)	119.395(4)	90	90
V [Å ^{3}]	6572(2)	3816(2)	5016(2)	3955.8(13)	6092(2)	12538(5)
Z	4	2	2	2	4	8
ρ_{calc} [gcm^{-3}]	0.967	1.160	1.110	1.120	1.085	1.014
Absorption coefficient [mm^{-1}]	0.244	0.315	0.369	0.720	0.314	0.256
F(000)	2096	1440	1816	1440	2168	4192
θ range	$1.73<\theta<26.36$	$1.18<\theta<25.00$	$1.25<\theta<25.00$	$1.42<\theta<25.50$	$1.37<\theta<26.37$	$1.60<\theta<25.00$
Reflections collected/unique	51743/13354	26562/13238	36177/17515	29478/14514	47608/13439	43762/11026
Completeness to θ [%]	99.6	98.7	99.2	98.7	99.8	99.9
Data/restraints/parameters	13350/0/541	13238/75/911	17515/6/907	14514/12/752	12439/0/556	11026/0/541
Goodness of fit on F^2	1.26	1.28	1.01	0.99	1.02	1.36
Final R indices [$I>2\sigma(I)$]	R1=0.070, *w*R2=0.143	R1=0.144, *w*R2=0.307	R1=0.143, *w*R2=0.260	R1=0.083, wR2=0.169	R1=0.085, *w*R2=0.165	R1=0.138, *w*R2=0.264
R indices (all data)	R1=0.076, *w*R2=0.146	R1=0.152, *w*R2=0.311	R1=0.165, *w*R2=0.280	R1=0.142, wR2=0.190	R1=0.095, *w*R2=0.171	R1=0.154, *w*R2=0.278
Largest diff. Peak/hole [e$^-$/ Å^3]	0.54/−0.32	1.01/−0.65	0.93/−0.46	0.76/−0.32	0.54/−0.34	0.60/−0.47

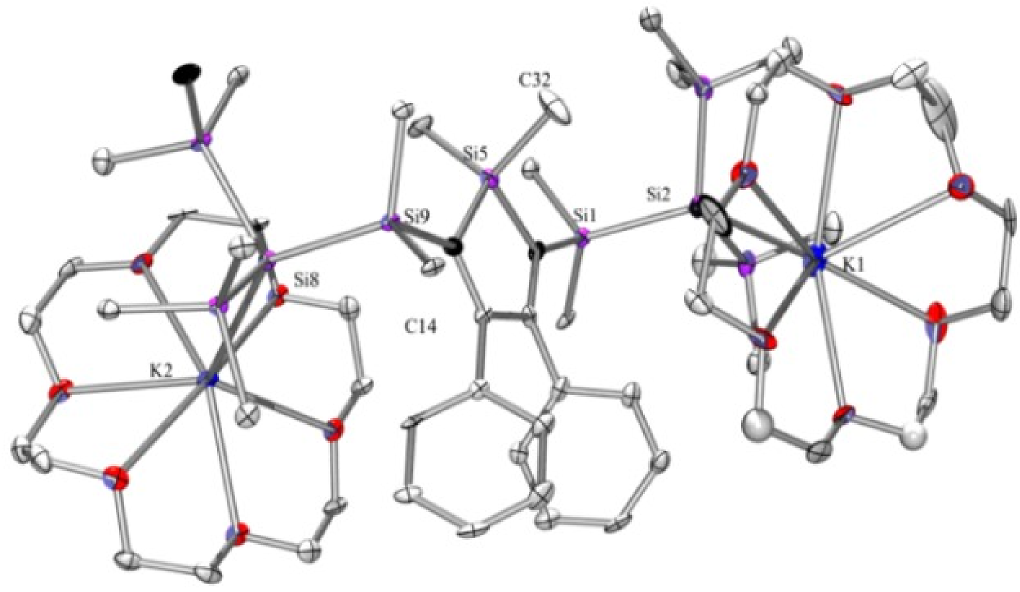

Figure S1. Molecular structure of **[K(18-crown-6)]$_2$[3a]** (thermal ellipsoid plot drawn at the 30% probability level). All hydrogen atoms are omitted for clarity (bond lengths in Å, angles in deg). Si(1)-C(15) 1.875(14), Si(1)-C(24) 1.881(15), Si(1)-C(23) 1.911(16), Si(1)-Si(2) 2.357(6), Si(2)-Si(3) 2.338(6), Si(2)-Si(4) 2.345(7), Si(2)-K(1) 3.467(5).

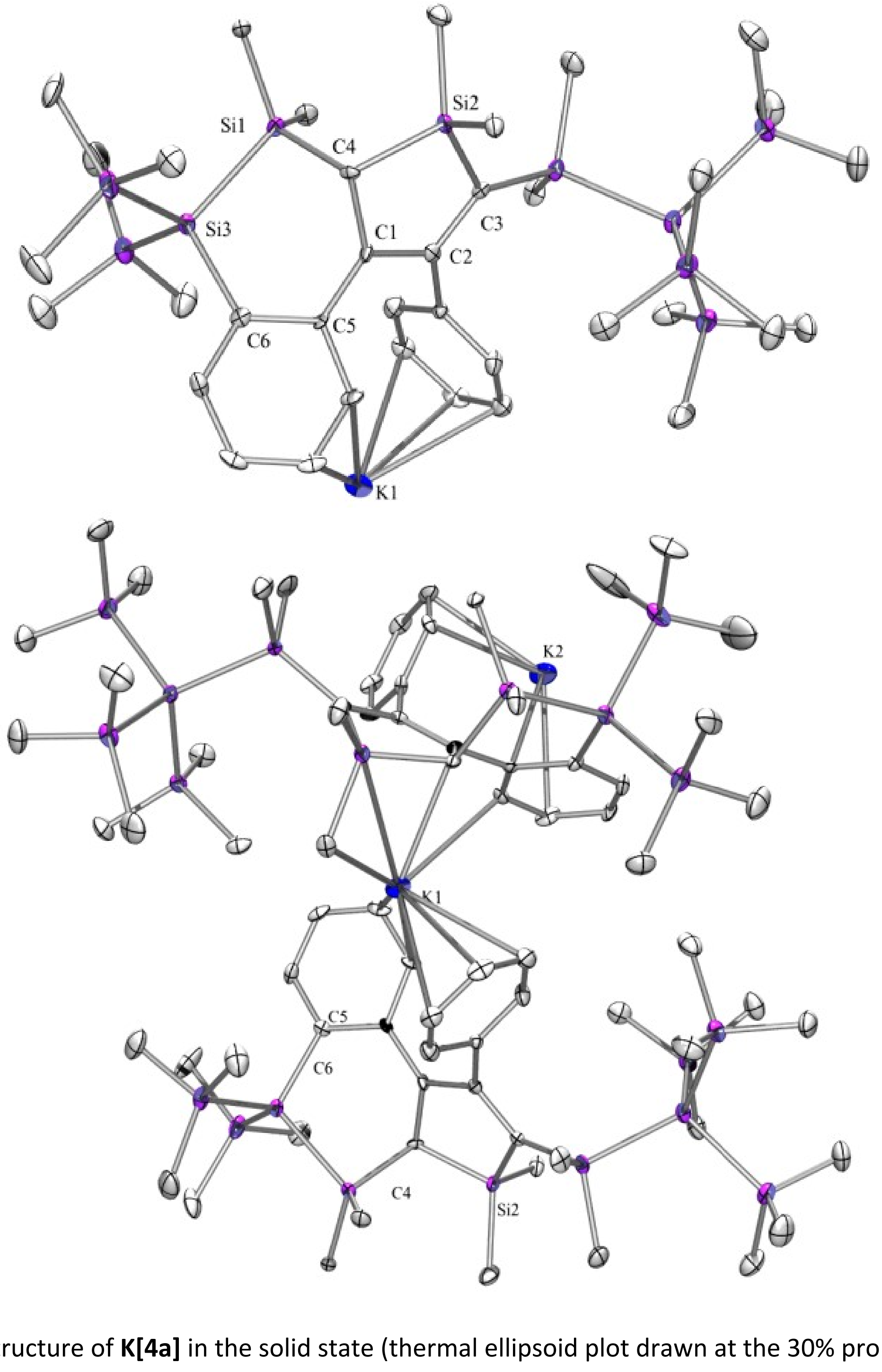

Figure S2. Structure of **K[4a]** in the solid state (thermal ellipsoid plot drawn at the 30% probability level). Top: one molecular subunit of the tetrameric cluster **$\{K[4a]\}_4$** Bottom: Connectivity between two of the four subunits of **$\{K[4a]\}_4$**. All hydrogen atoms are omitted for clarity (bond lengths in Å, angles in deg). Si(1)-C(4) 1.868(8), Si(1)-Si(3) 2.334(3), Si(2)-C(3) 1.860(8), Si(2)-C(4) 1.893(8), Si(3)-Si(8) 2.340(3), Si(4)-C(3) 1.843(8), C(5)-C(1) 1.425(11), C(5)-C(6) 1.455(11), C(1)-C(2) 1.445(11), C(1)-C(4) 1.524(10), C(2)-C(3) 1.401(11).

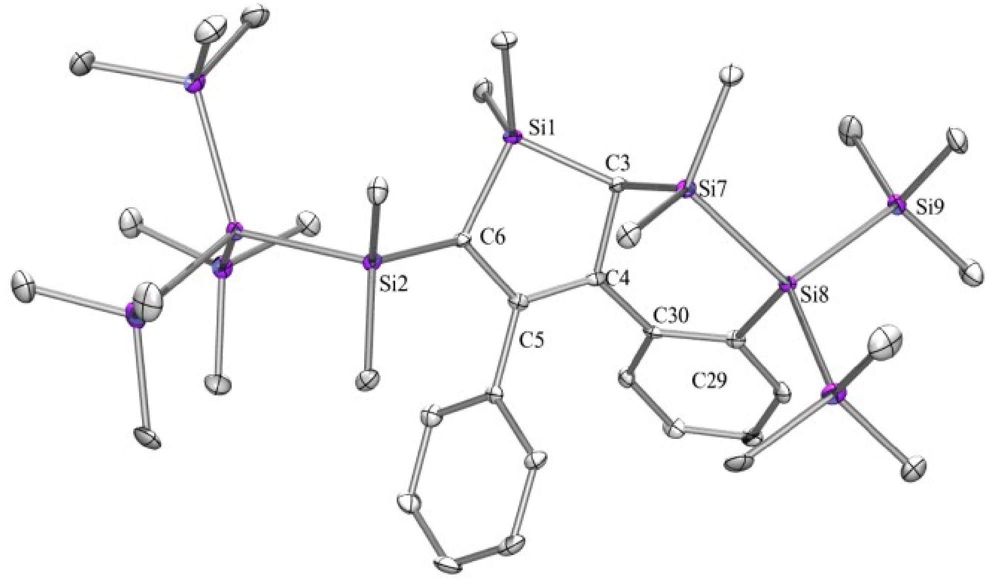

Figure S3. Molecular structure of the anionic part of **[BrMg(THF)$_5$][4a]**, with the [BrMg(THF)$_5$]$^+$ unit, a THF molecule and hydrogen atoms omitted for clarity (thermal ellipsoid plot drawn at the 30% probability level, bond lengths in Å, angles in deg). C(6)-Si(2) 1.820(6), Si(2)-Si(3)-2.410(2), Si(3)-Si4 2.353(3), Si(3)-Si(6) 2.351(4), Si(7)-Si(8) 2.342(2), Si(8)-Si(9) 2.341(3), Si(8)-Si(10) 2.338(3), Si(1)-C(6) 1.825(8), Si(1)-C(3) 1.895(5), C(3)-Si7 1.87(1), C(3)-C(4) 1.54(1), C(4)-C(5)-1.379(8), C(5)-C(6) 1.448(8), Si(8)-C(29) 1.90(1), C(30)-C(4) 1.474(8), Si(1)-C(3)-Si(7) 117.2(4), C(4)-C(3)-Si(7) 107.2(5), Si(1)-C(3)-C(4) 100.0(5), Si(10)-Si(8)-Si(9) 111.6(1), Si(3)-Si(2)-C(6) 121.3(3).

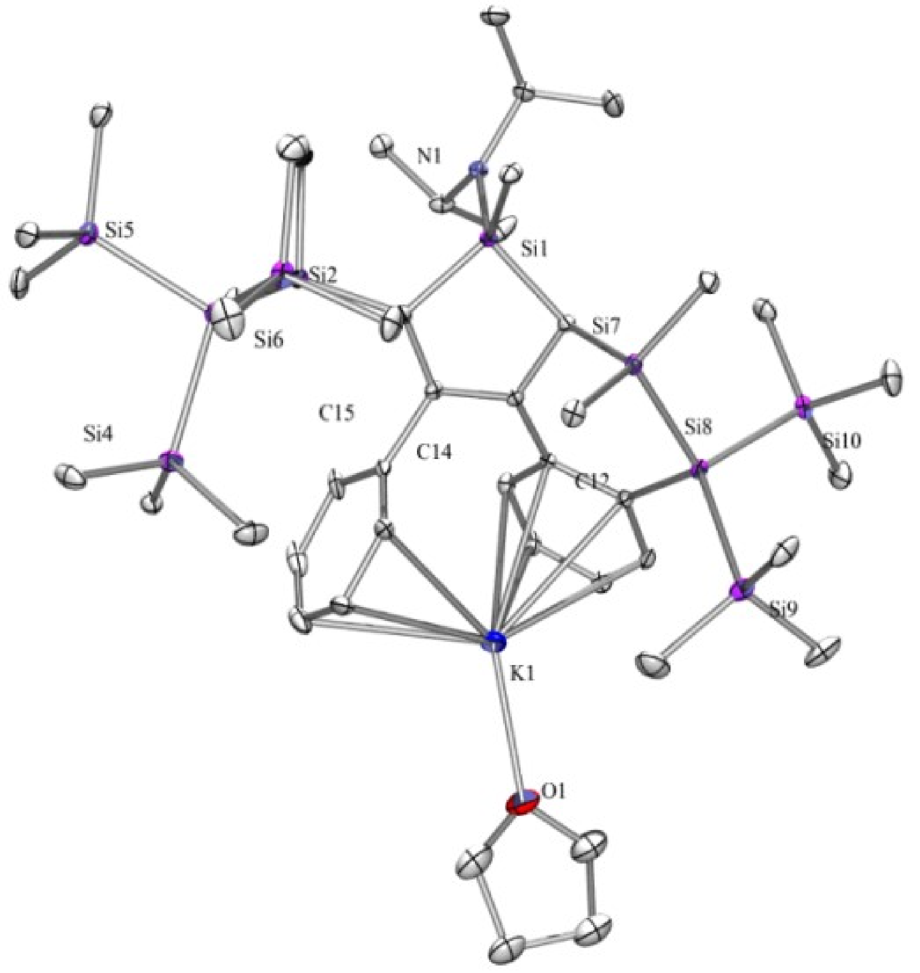

Figure S4. Molecular structure of **[K(THF)][4b]** (thermal ellipsoid plot drawn at the 30% probability level). All hydrogen atoms are omitted for clarity (bond lengths in Å, angles in deg). Si(1)-N(1) 1.753(3), Si(2)-Si(3) 2.4136(15), Si(3)-Si(6) 2.3441(15), Si(3)-Si(4) 2.3523(15), Si(3)-Si(5) 2.3619(15), Si(7)-Si(8) 2.3310(15), Si(8)-C(12) 1.896(4), Si(8)-Si(9) 2.3403(15), Si(8)-Si(10) 2.3450(15), K(1)-O(1) 2.601(4), C(14)-C(15) 1.414(5), N(1)-Si(1)-C(16) 111.99(16), Si(6)-Si(3)-Si(4) 106.79(6), Si(6)-Si(3)-Si(5) 108.04(6), Si(4)-Si(3)-Si(5) 103.66(6), Si(6)-Si(3)-Si(2) 110.19(6), Si(4)-Si(3)-Si(2) 117.49(6), Si(5)-Si(3)-Si(2) 110.14(6), C(15)-C(14)-C(11) 126.1(3).

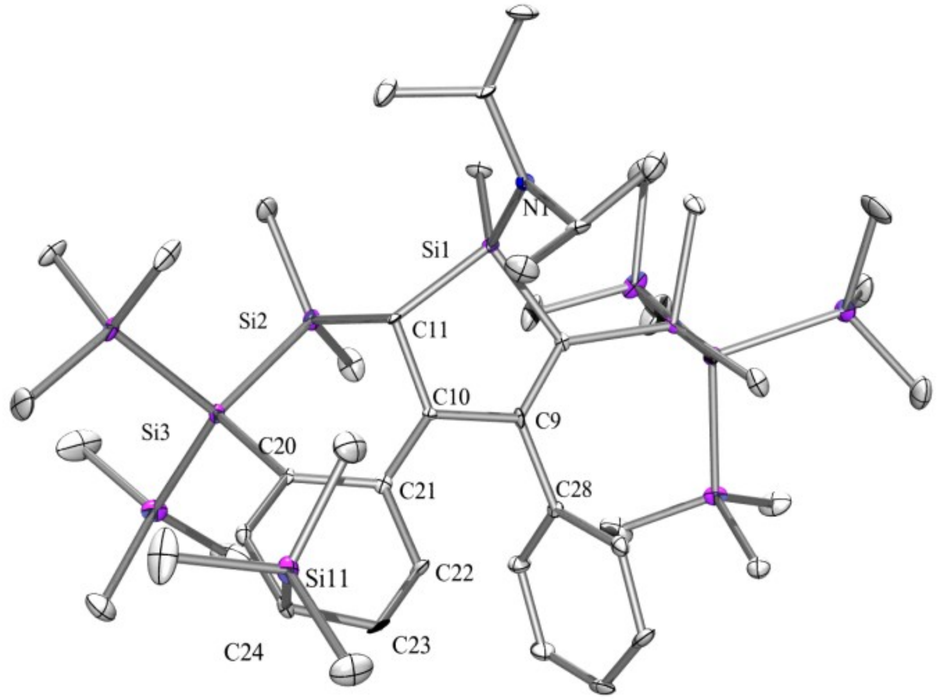

Figure S5. Molecular structure of **5b** (thermal ellipsoid plot drawn at the 30% probability level). All hydrogen atoms are omitted for clarity (bond lengths in Å, angles in deg). Si(1)-N(1) 1.732(6), Si(1)-C(11) 1.880(7), Si(2)-Si(3) 2.325(3), Si(3)-Si(5) 2.338(3), Si(3)-Si(4) 2.352(3), Si(7)-Si(10) 2.347(3), Si(7)-Si(8) 2.357(3), Si(7)-Si(9) 2.373(3), Si(11)-C(24) 1.897(7), N(1)-C(6) 1.476(9), C(20)-C(21) 1.488(9), C(21)-C(22) 1.448(10), C(22)-C(23) 1.332(10), C(23)-C(24) 1.492(10).

3. ^{1}H, ^{13}C, and ^{29}Si NMR spectra

Compound **1b**: ^{1}H, ^{13}C{^{1}H}, and ^{29}Si{^{1}H} NMR spectra:

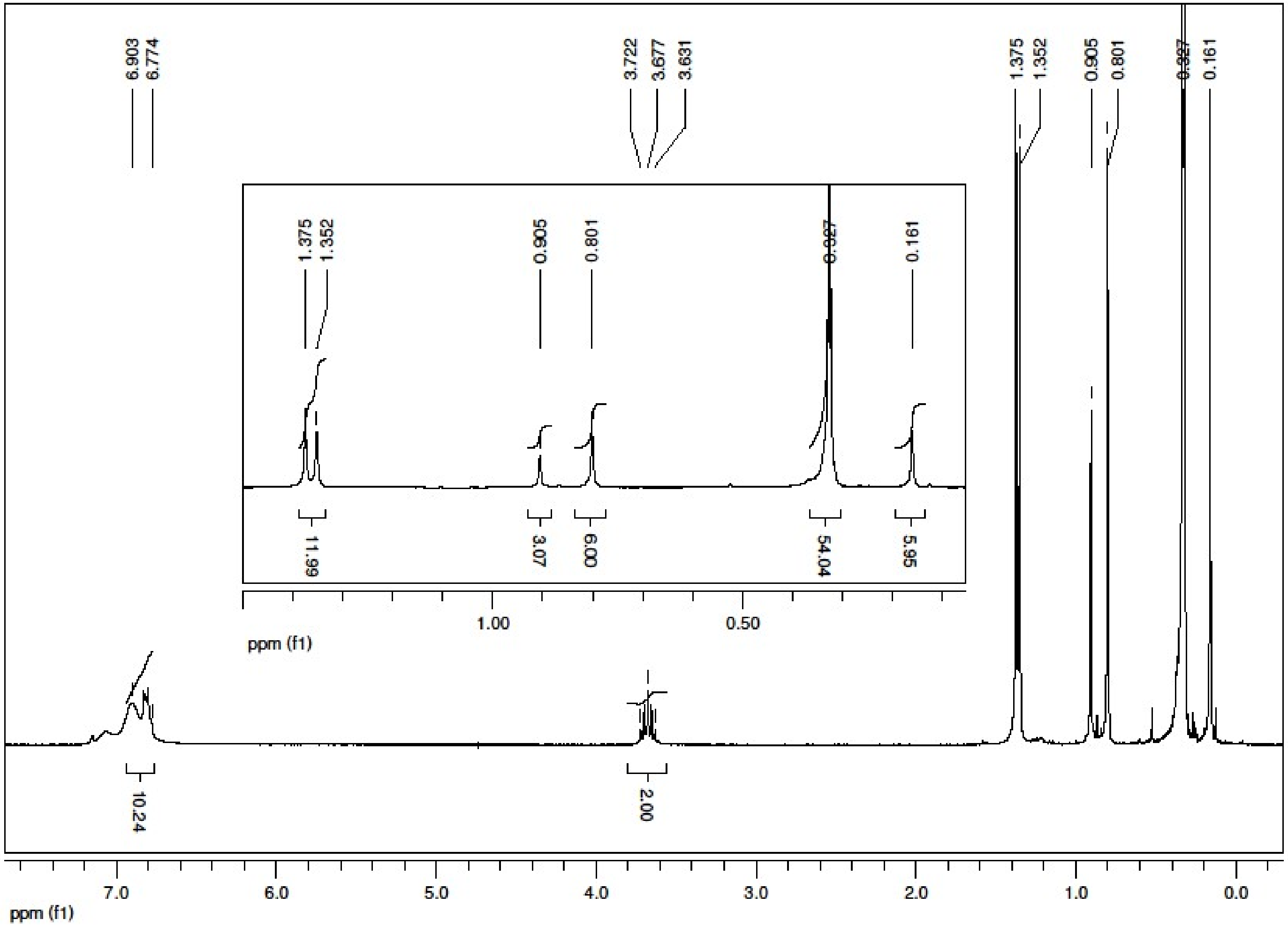

Figure S6. ^{1}H NMR spectrum of **1b** in C_6D_6.

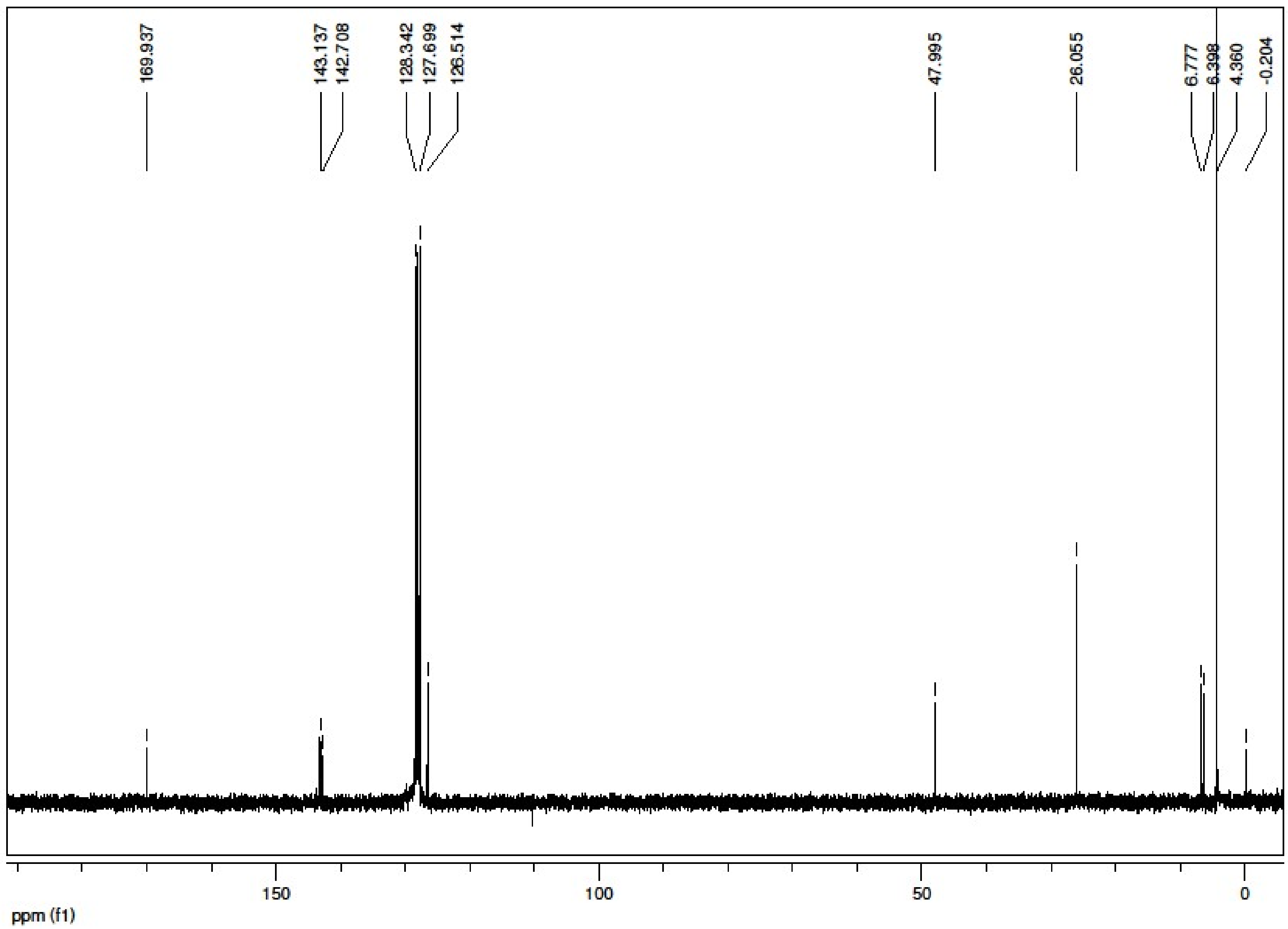

Figure S7. $^{13}C\{^{1}H\}$ NMR spectrum of **1b** in C_6D_6.

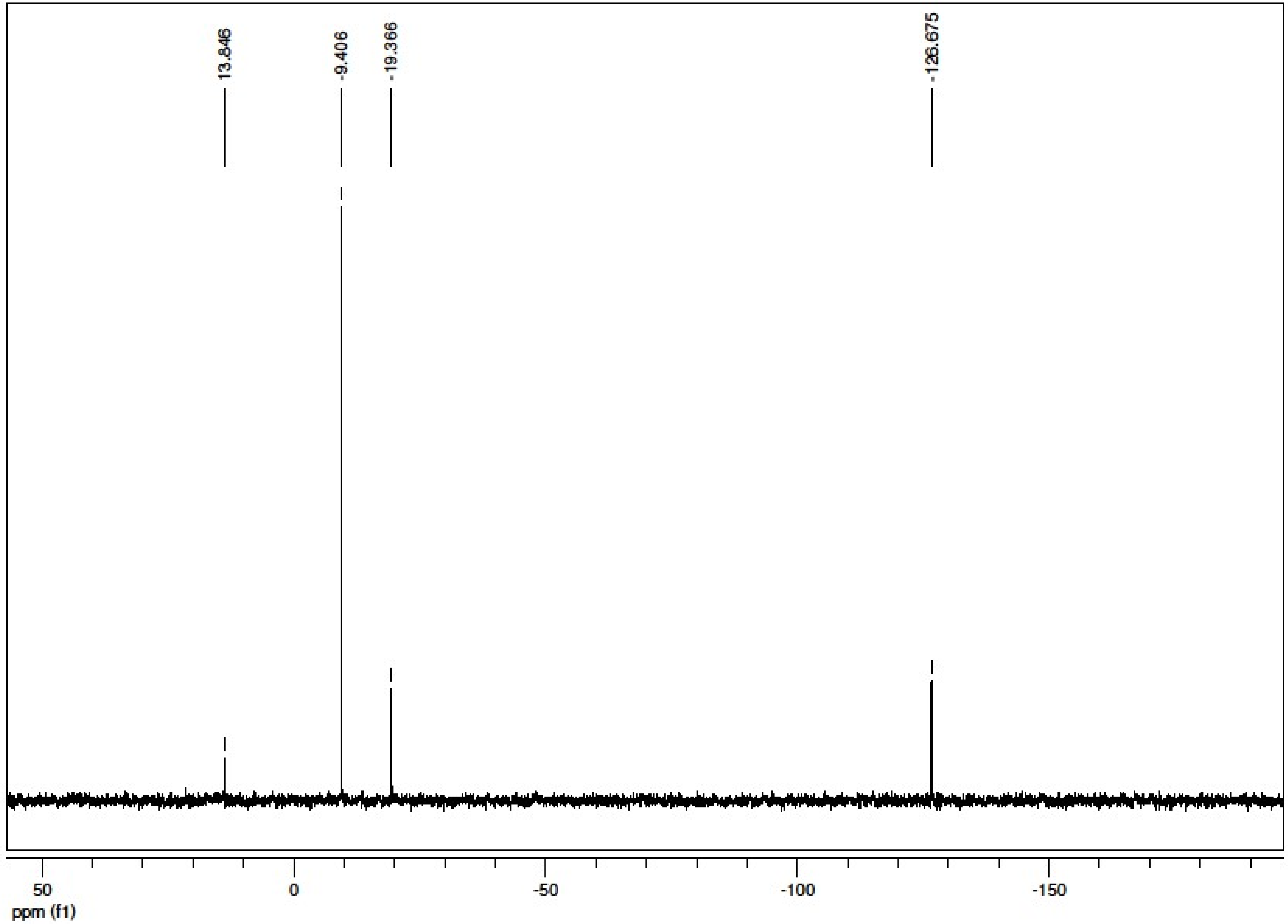

Figure S8. $^{29}Si\{^1H\}$ INEPT NMR spectrum of **1b** in C_6D_6.

Compound **[K(18-crown-6)]$_2$[3a]**: ^{1}H, ^{13}C{^{1}H}, and ^{29}Si{^{1}H} NMR spectra:

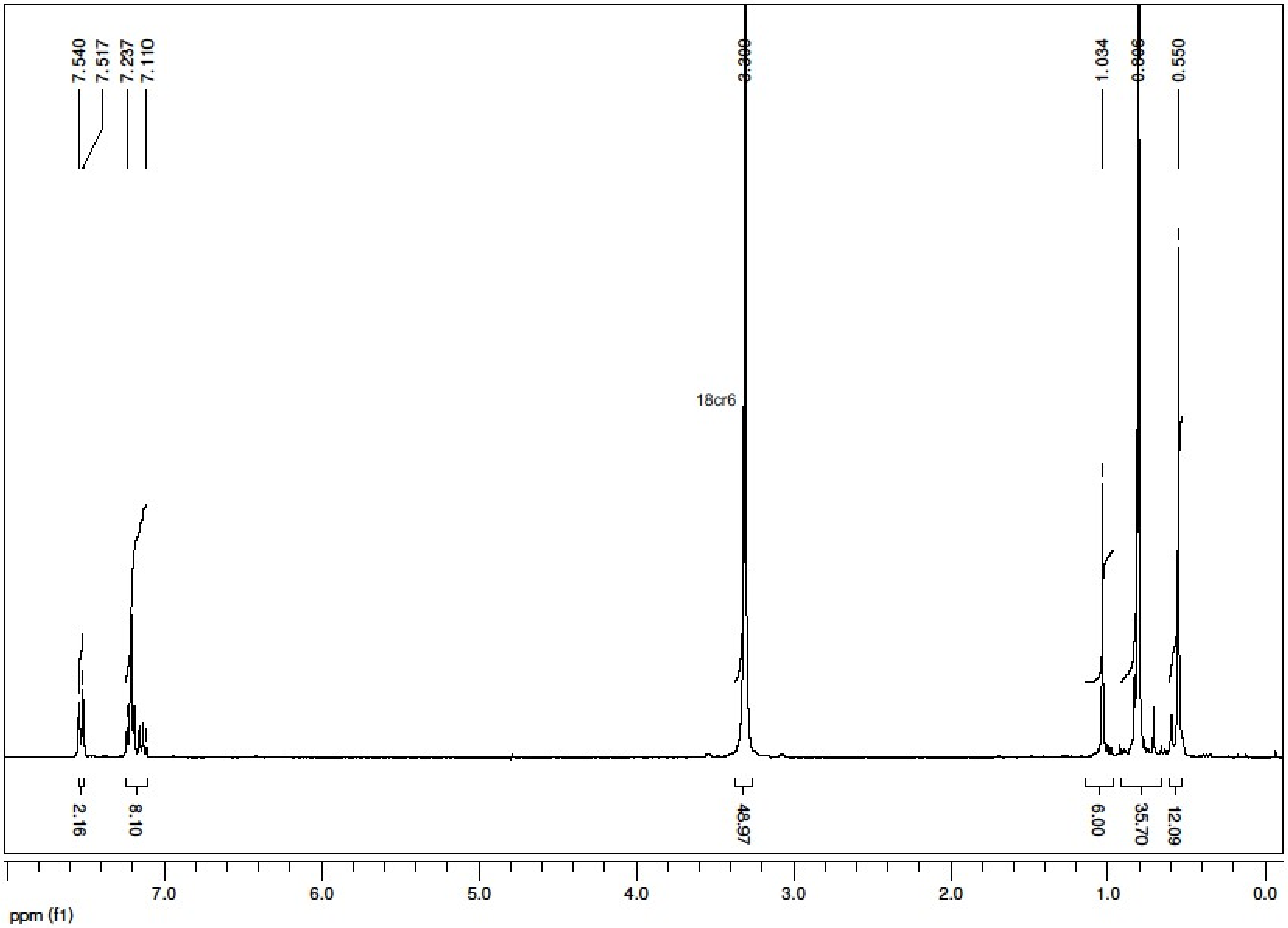

Figure 9. ^{1}H NMR spectrum of **[K(18-crown-6)]$_2$[3a]** in C_6D_6.

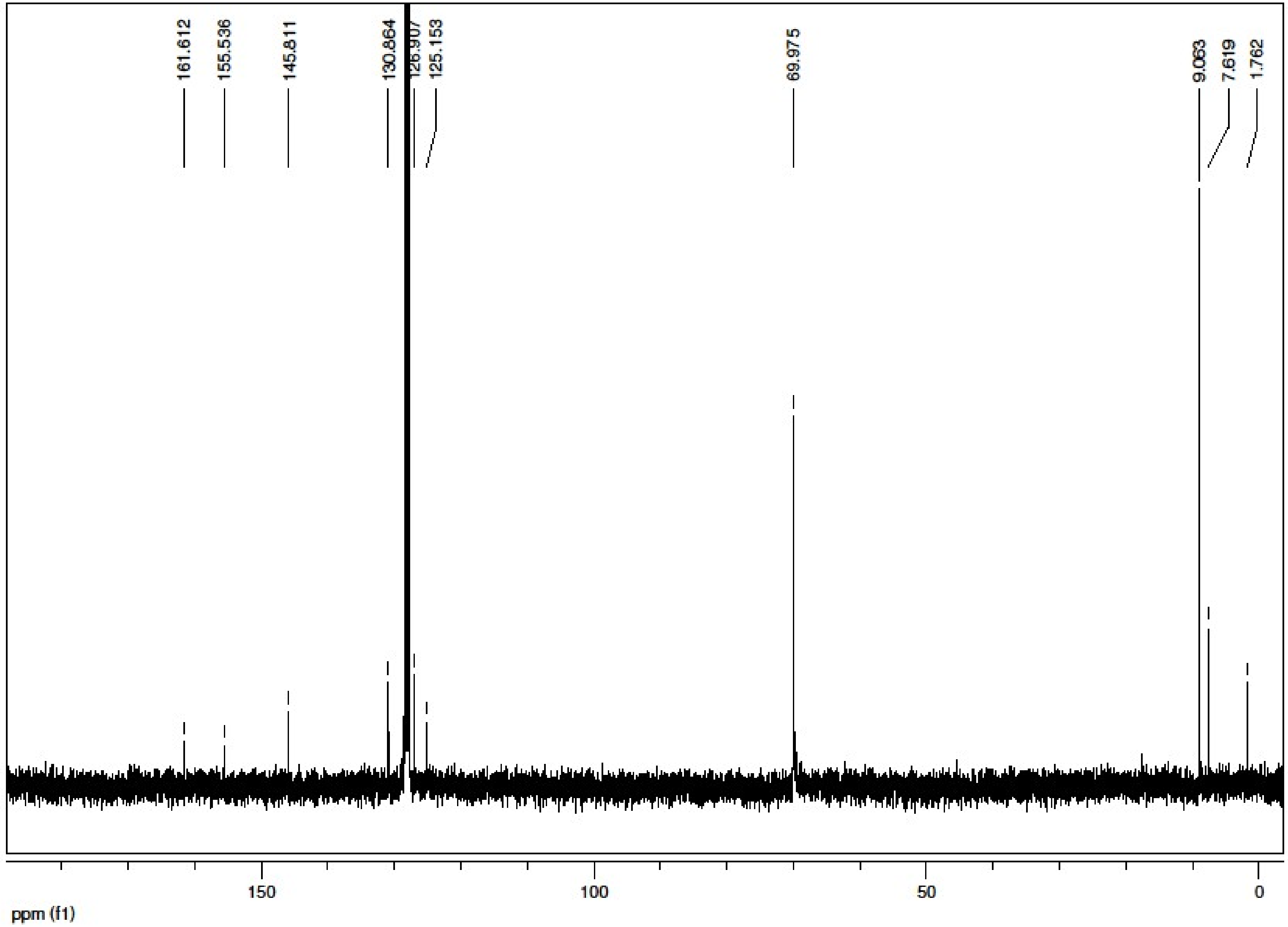

Figure S10. $^{13}C\{^{1}H\}$ NMR spectrum of **[K(18-crown-6)]$_2$[3a]** in C_6D_6.

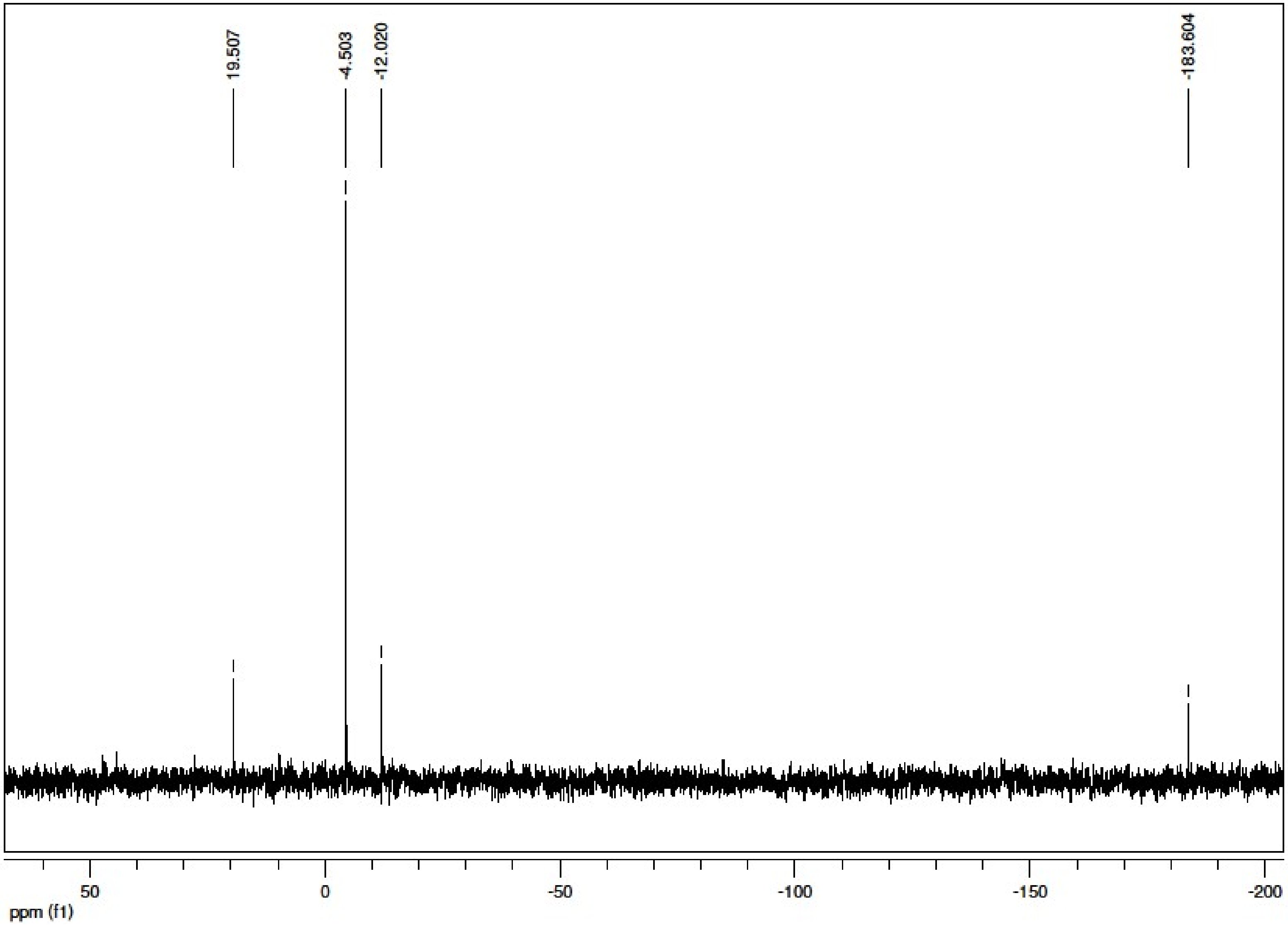

Figure S11. $^{29}Si\{^1H\}$ INEPT NMR spectrum of **[K(18-crown-6)]$_2$[3a]** in C_6D_6.

Compound **[K(18-crown-6)]$_2$[3b]**: ^{1}H, ^{13}C{^{1}H}, and ^{29}Si{^{1}H} NMR spectra:

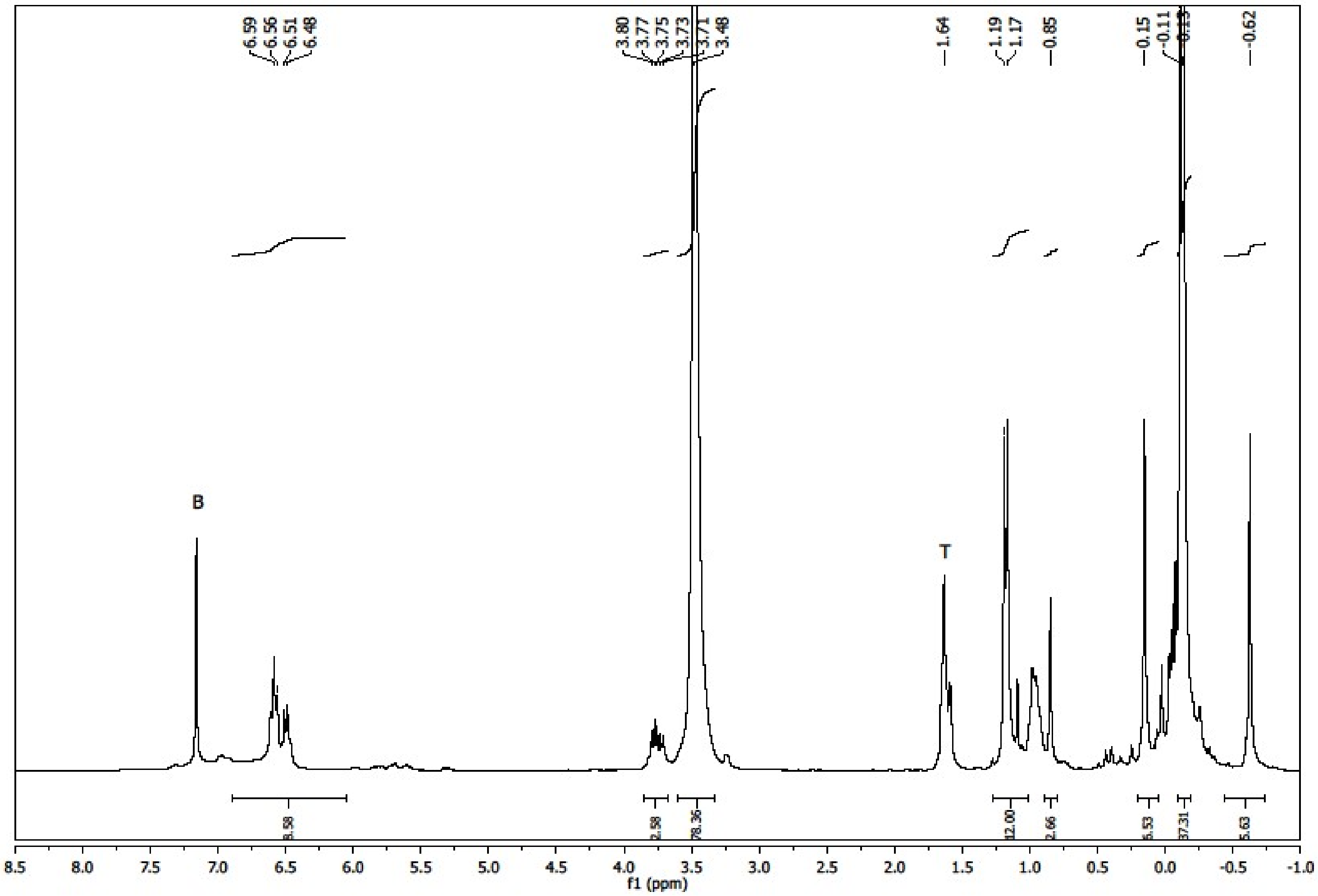

Figure S12. ^{1}H NMR spectrum of **K$_2$[3b]** in d8-THF, THF (T), benzene (B).

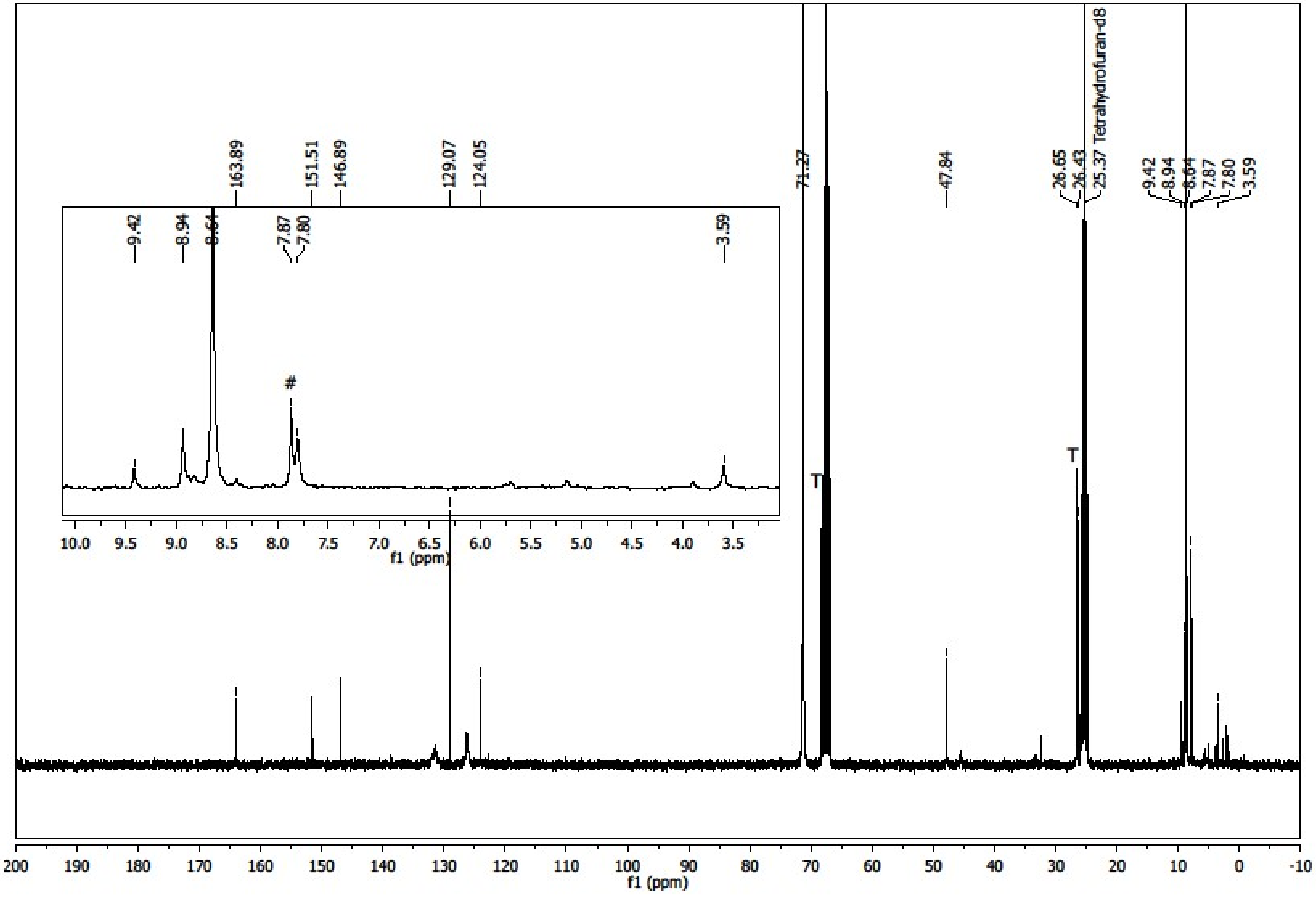

Figure S13. $^{13}C\{^{1}H\}$ NMR spectrum of **[K(18-crown-6)]$_2$[3b]** in d8-THF), $(Me_3Si)_3SiK(18cr6)$ (#), THF (T).

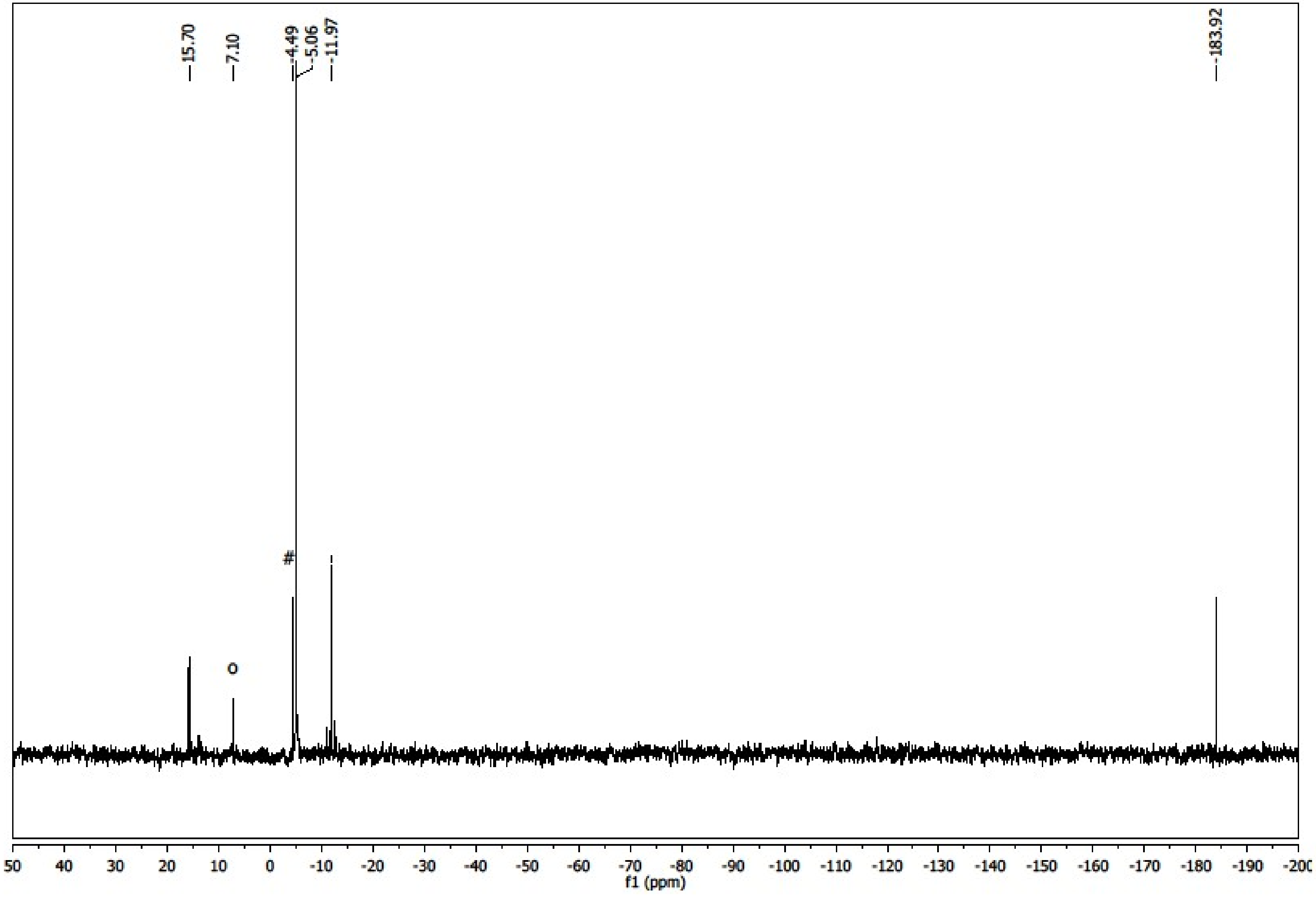

Figure S14. $^{29}Si\{^{1}H\}$ INEPT NMR spectrum of **[K(18-crown-6)]$_2$[3b]** in d8-THF. Me_3SiO^tBu (o), $(Me_3Si)_3SiK$(18cr6) (#).

Compound **K[4a]**: ^{1}H, $^{13}C\{^{1}H\}$, and $^{29}Si\{^{1}H\}$ NMR spectra:

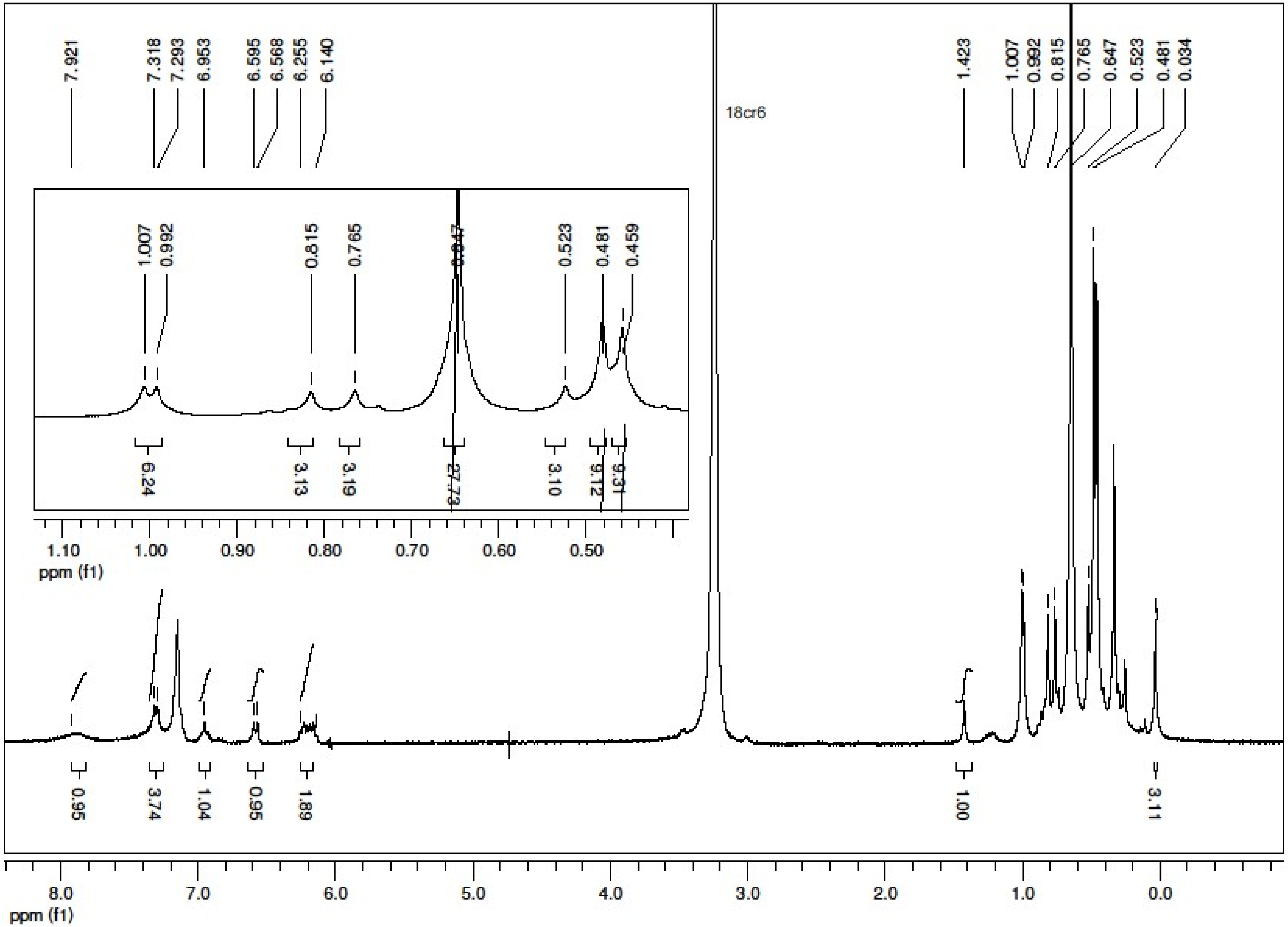

Figure S15. ^{1}H NMR spectrum of **K[4a]** in C_6D_6.

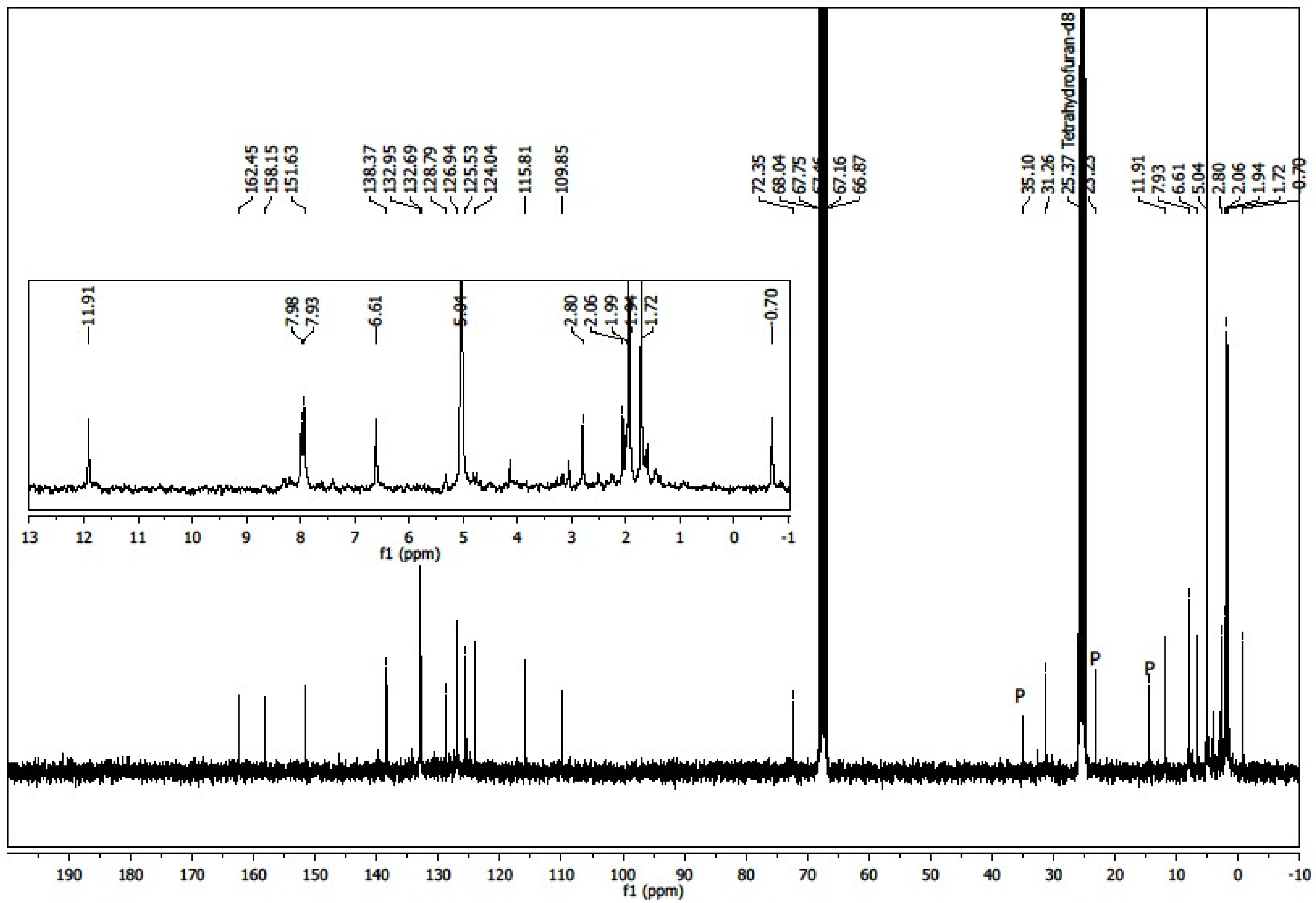

Figure S16. $^{13}C\{^{1}H\}$ NMR spectrum of **K[4a]** in d8-THF (cocrystallized pentane labeled with P).

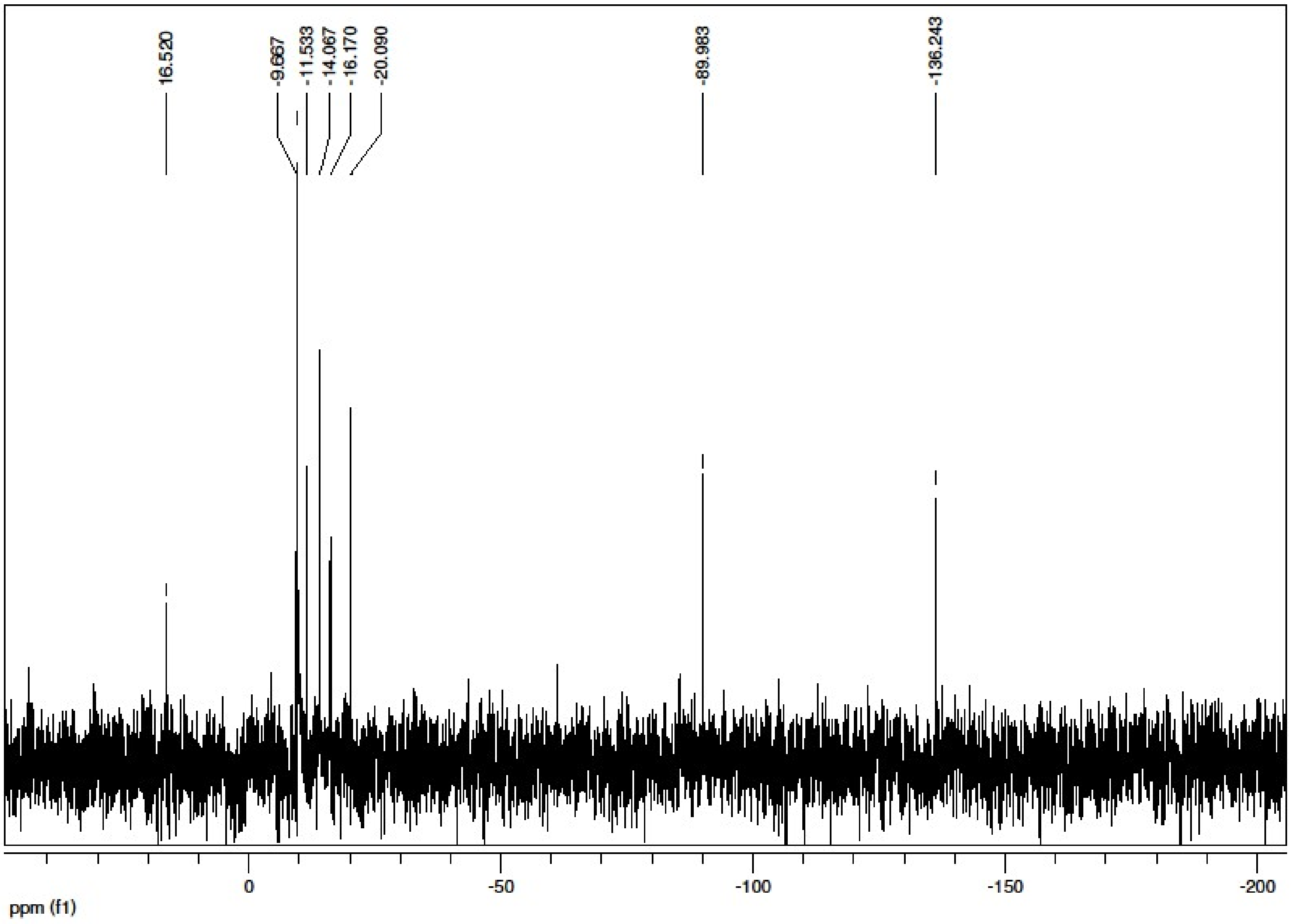

Figure S17. $^{29}Si\{^{1}H\}$ INEPT NMR spectrum of **K[4a]** in C_6D_6.

Compound **K[4b]**: ^{1}H, $^{13}C\{^{1}H\}$, and $^{29}Si\{^{1}H\}$ NMR spectra:

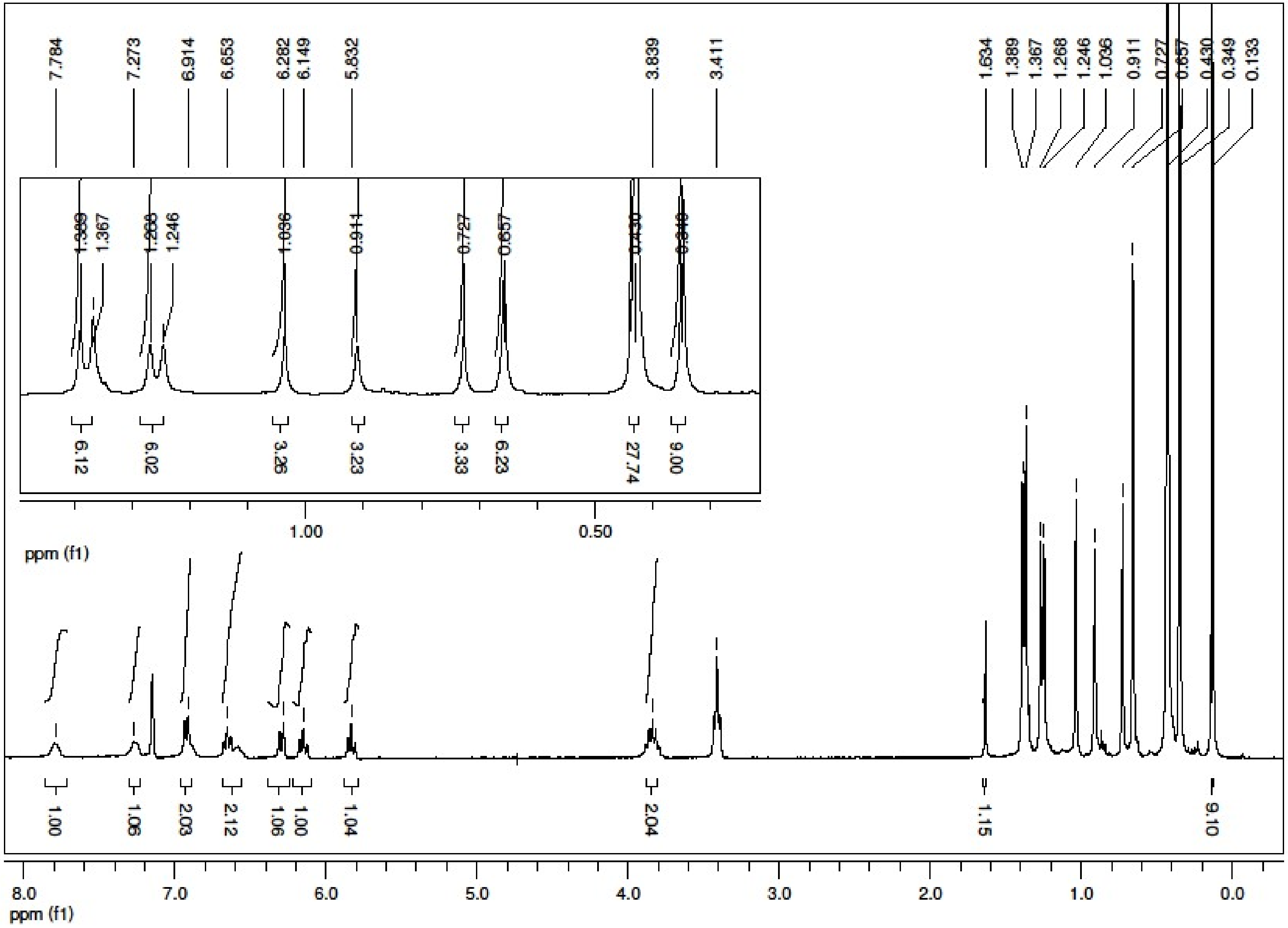

Figure S18. ^{1}H NMR spectrum of **[K(THF)][4b]** in C_6D_6.

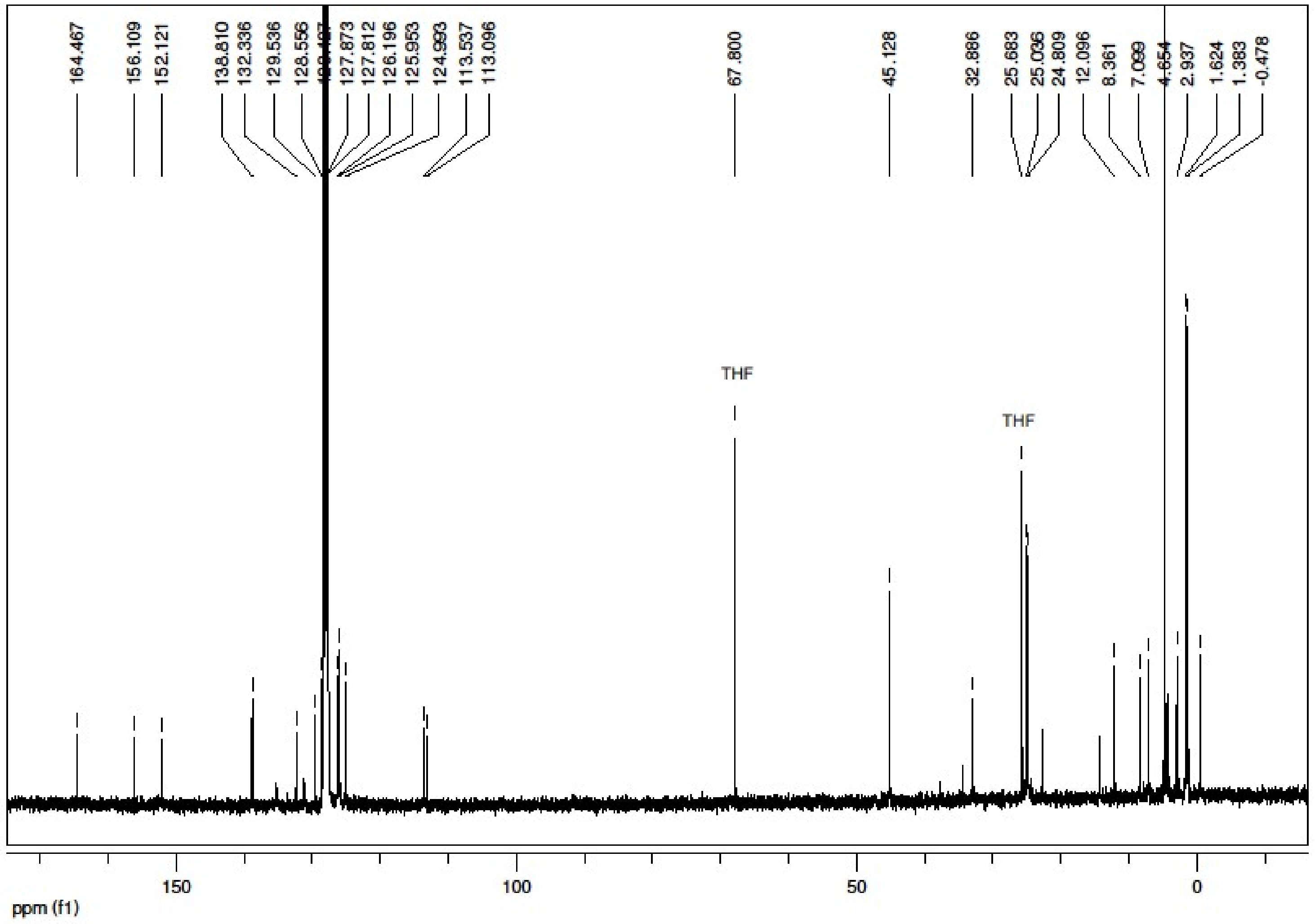

Figure S19. $^{13}C\{^{1}H\}$ NMR spectrum of **[K(THF)][4b]** in C_6D_6.

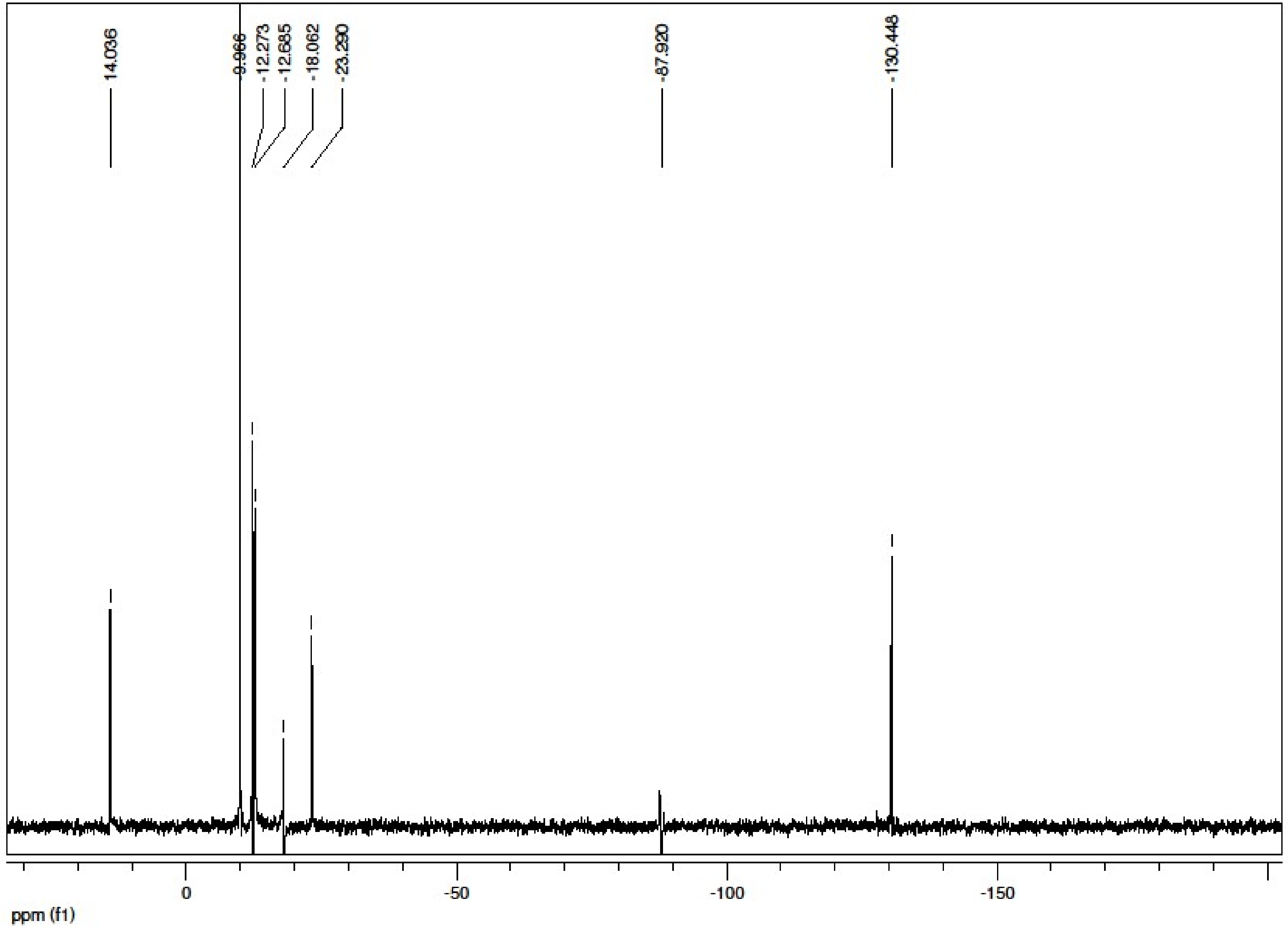

Figure S20. $^{29}Si\{^1H\}$ INEPT NMR spectrum of **[K(THF)][4b]** in C_6D_6.

Compound **5b**: ^{1}H, $^{13}C\{^{1}H\}$, and $^{29}Si\{^{1}H\}$ NMR spectra:

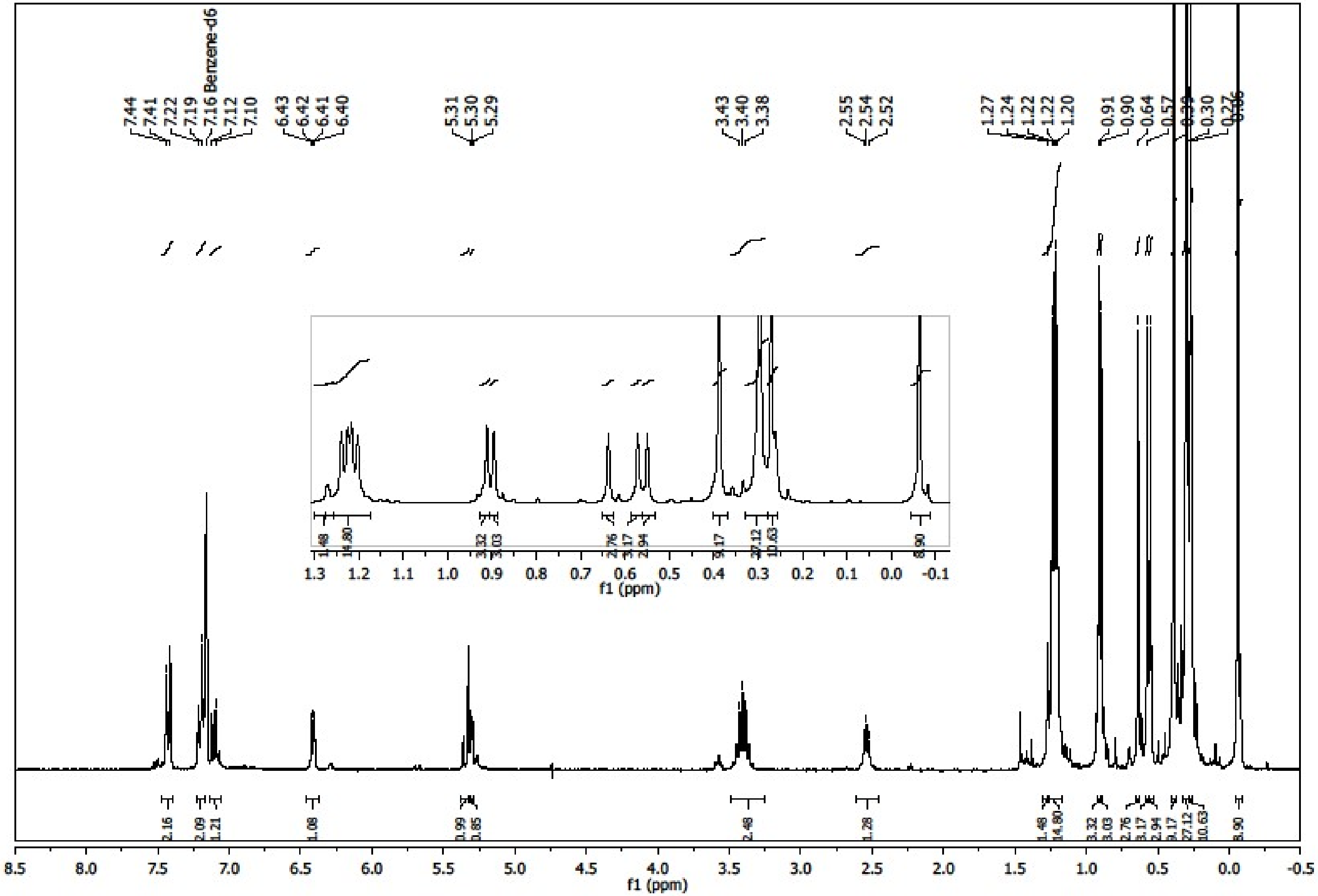

Figure S21. ^{1}H NMR spectrum of **5b** in C_6D_6.

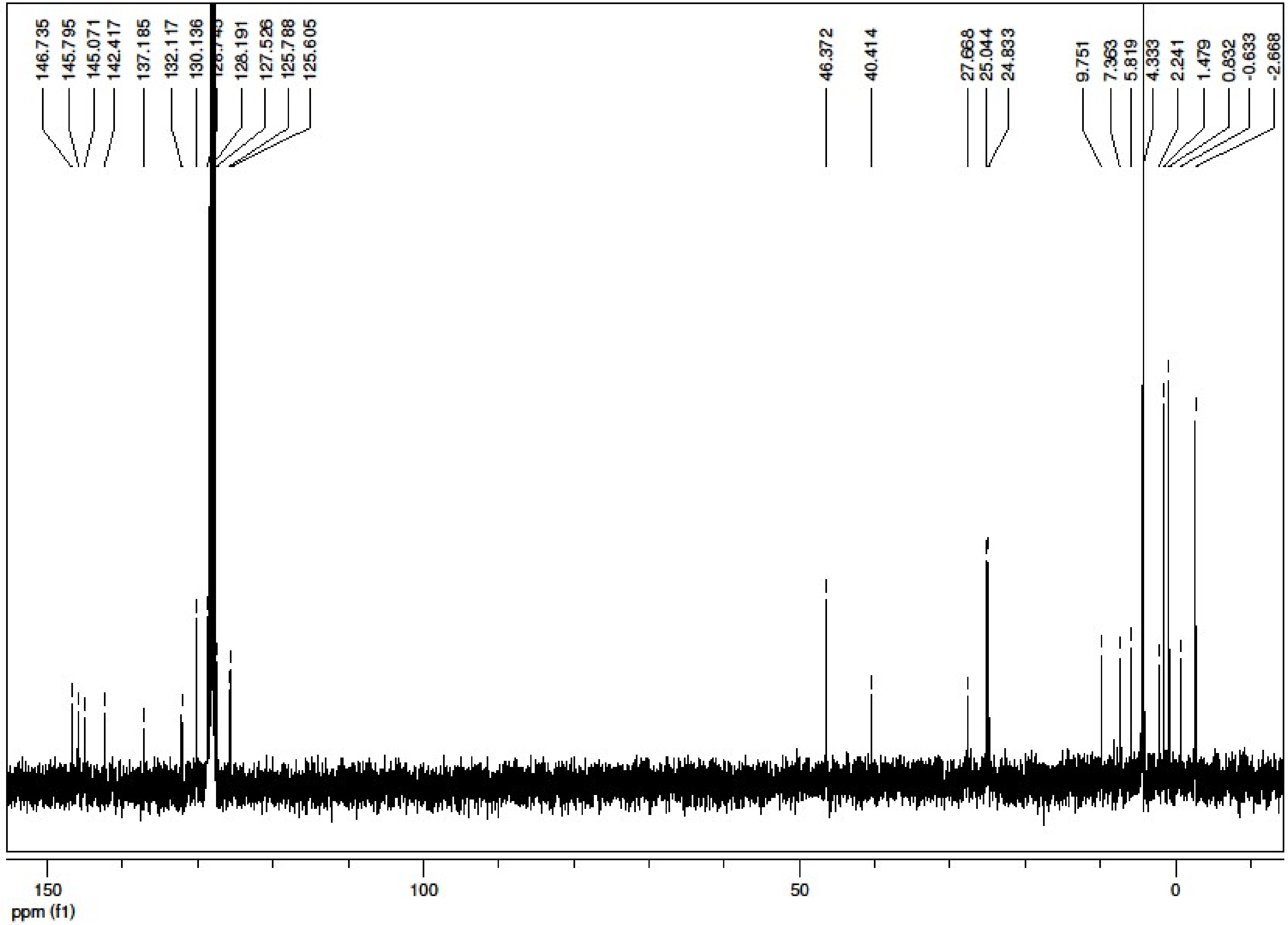

Figure S22. $^{13}C\{^1H\}$ NMR spectrum of **5b** in C_6D_6.

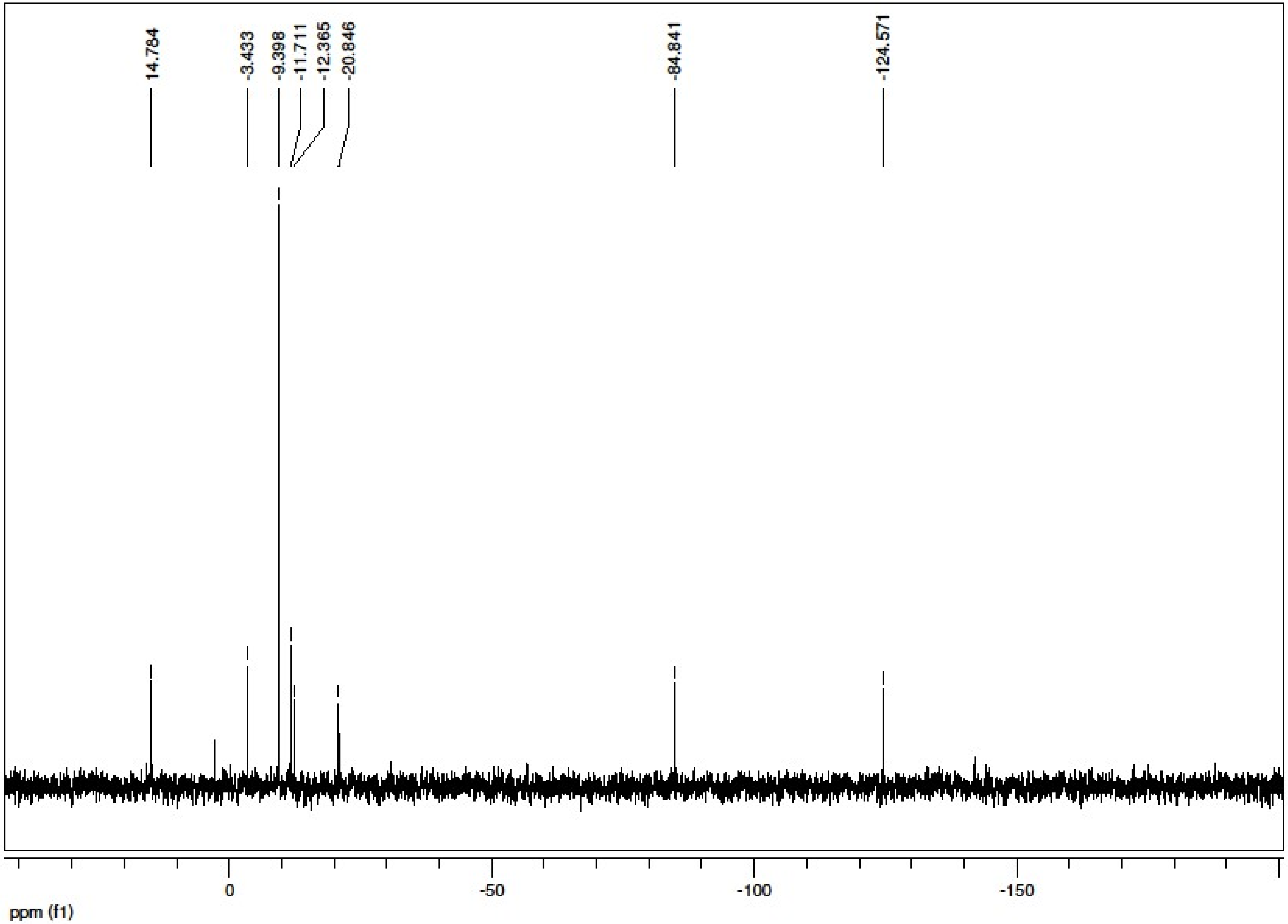

Figure S23. $^{29}Si\{^{1}H\}$ INEPT NMR spectrum of **5b** in C_6D_6.

4. Computational details

All quantum chemical calculations were carried out using the Gaussian16 package.[S16]
The molecular structure optimizations were performed using the M06-2X functional[S17] along with the def2-TZVP basis set for all elements.[S18,S19] Every stationary point was identified by a subsequent frequency calculation either as minimum (Number of imaginary frequencies (NIMAG) = 0) or transition state (TS) (NIMAG = 1). Intrinsic Reaction Coordinate (IRC)-type of calculations verified the connections between transition states and corresponding minima.[S20] The SCF energies, (E(SCF)) at the M06-2X/def2-TZVP level and the absolute computed Gibbs free energies at T = 298.15 K and p = 0.101 MPa (1 atm) in the gas phase (G298) are given in Tables S3-S5 for all optimized molecular structures.

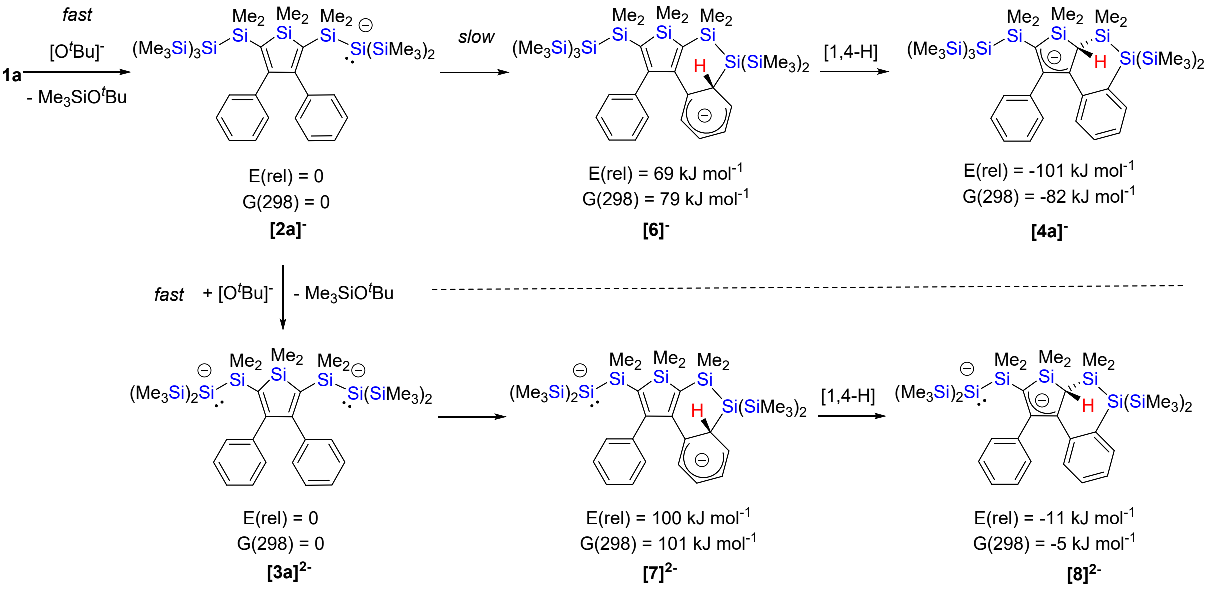

Scheme S1. Top: Suggested reaction mechanism for the formation of allylic anion [**4a**]$^-$ from the silyl anion [**2a**]$^-$. Bottom: Corresponding reaction path for the dianion [**3a**]$^{2-}$. Relative energies, E(rel), and Gibbs enthalpies at 298K G(298) are calculated at the M06-2X/def2-TZVP level.

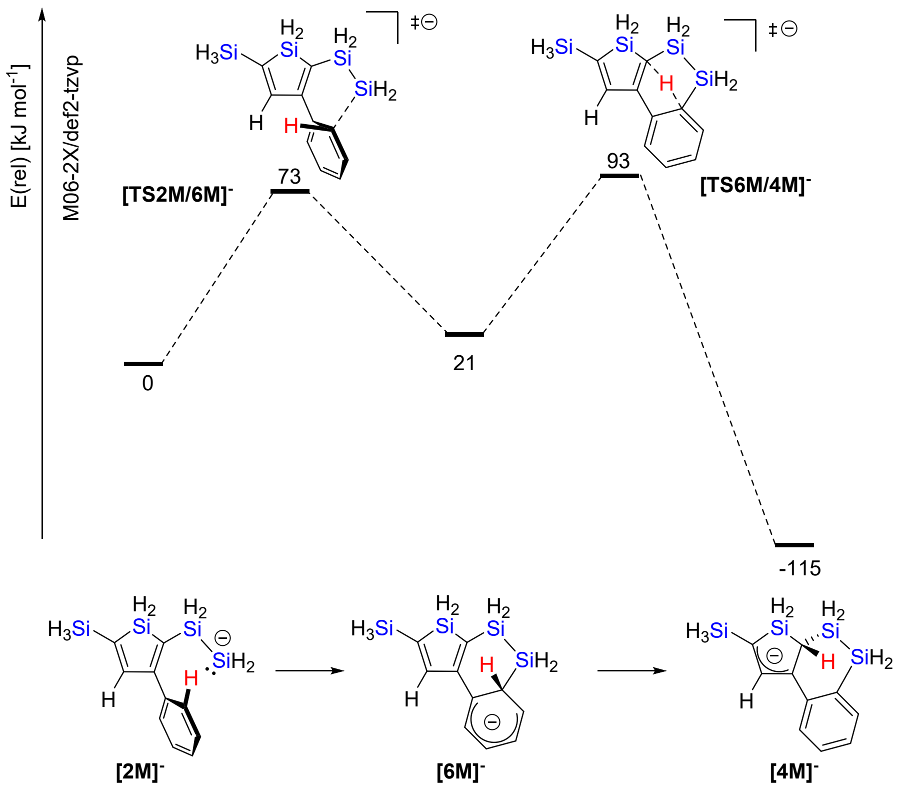

Figure S24. Calculated reaction coordinate for the formation of the model anion [**4M**]⁻ from the silyl anion [**2M**]⁻. Energies relative to [**2M**]⁻ are calculated at the M06-2X/def2-TZVP level.

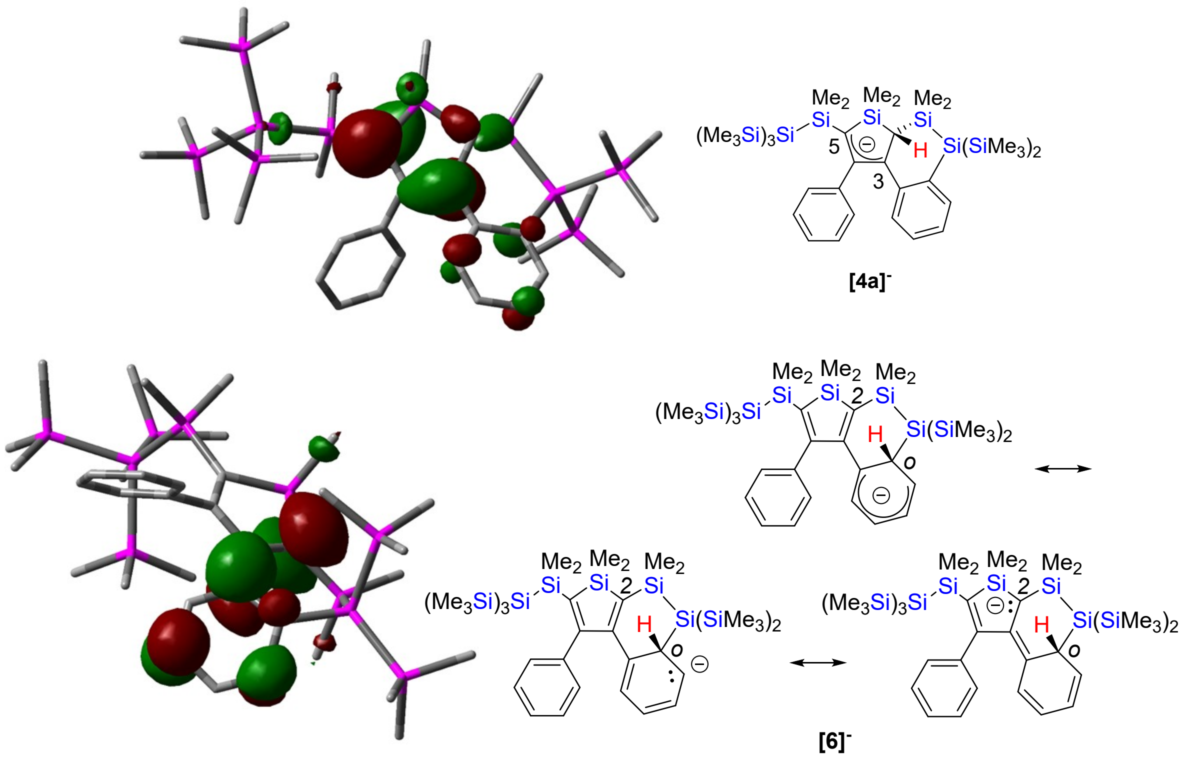

Figure 25. Calculated surface diagrams of the HOMO of allylic anion [**4a**]⁻ (top) and of the intermediate anion [**6**]⁻ (bottom) (M06-2X/def2-TZVP level, isodensity value 0.04). Color code: violet Si, grey C, Hydrogen atoms are not shown.

Table S2. Absolute SCF energies, (E(SCF)) at the M06-2X/def2-TZVP level and absolute computed Gibbs free energies at T = 298.15 K and p = 0.101 MPa (1 atm) in the gas phase (G298) for compounds of Scheme S1.

	E [a.u.]	G [a.u.]	NIMAG	ZPVE [kJ mol^{-1}]
**$[2a]^-$**	-4348.90407	-4348.01258	0	2622
**$[6]^-$**	-4348.87794	-4347.98693	0	2617
**$[4a]^-$**	-4348.94257	-4348.05451	0	2617
**$[3a]^{2-}$**	-3939.62556	-3938.84486	0	2318
**$[7]^{2-}$**	-3939.58757	-3938.80639	0	2311
**$[8]^{2-}$**	-3939.62963	-3938.84689	0	2314

Table S3. Absolute SCF energies, (E(SCF)) at the M06-2X/def2-TZVP level and absolute computed Gibbs free energies at T = 298.15 K and p = 0.101 MPa (1 atm) in the gas phase (G^{298}) for compounds of Figure 9.

	E [a.u.]	G [a.u.]	NIMAG	$\tilde{\upsilon}$[cm^{-1}]	ZPVE [kJ mol^{-1}]
**$[2M]^-$**	-1547.97207	-1547.81547	0	/	5303
**$[TS2M/6M]^-$**	-1547.94449	-1547.78603	1	-381	5289
**$[6M]^-$**	-1547.96400	-1547.80428	0	/	5324
**$[TS6M/4M]^-$**	-1547.93659	-1547.78021	1	-1548	5208
**$[4M]^-$**	-1548.01603	-1547.85614	0	/	5337

Table S4. Absolute SCF energies, (E(SCF)) at the M06-2X/def2-TZVP level and absolute computed Gibbs free energies at T = 298.15 K and p = 0.101 MPa (1 atm) in the gas phase (G^{298}) for compounds of Figure 11.

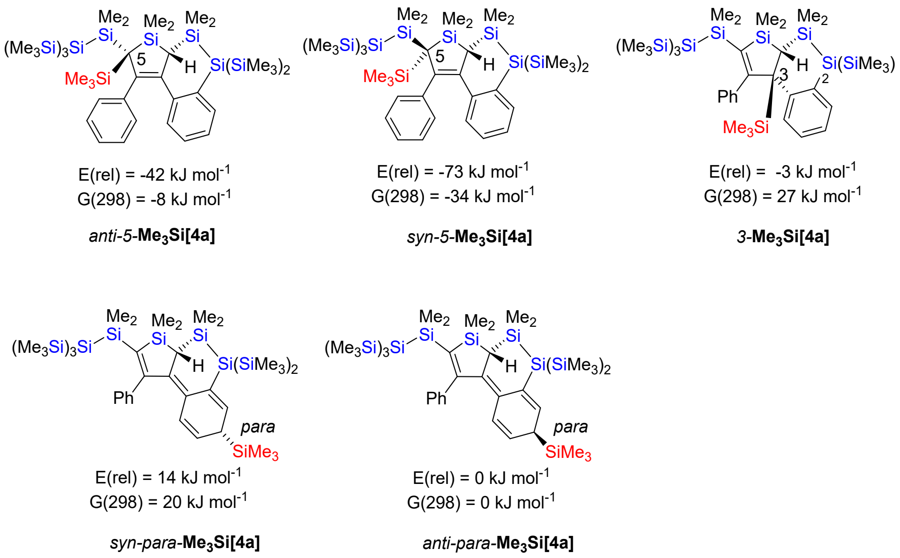

	E [a.u.]	G [a.u.]	NIMAG	ZPVE [kJ mol^{-1}]
***anti*-5-Me$_3$Si[4a]**	-4758.12809	-4757.11869	0	2937
***syn*-5-Me$_3$Si[4a]**	-4758.13999	-4757.12840	0	2938
**3-Me$_3$Si[4a]**	-4758.11321	-4757.10518	0	2934
***anti-para*-Me$_3$Si[4a]**	-4758.11215	-4757.11561	0	2922
***syn-para*-Me$_3$Si[4a]**	-4758.10694	-4757.10789	0	2924

5. References

S1 Pangborn, A. B.; Giardello, M. A.; Grubbs, R. H.; Rosen, R. K.; Timmers, F. J. Safe and Convenient Procedure for Solvent Purification. *Organometallics* **1996**, *15*, 1518–1520.

S2 Huppmann, F.; Maringgele, W.; Kottke, T.; Meller, A. Reaktionen subvalenter Verbindungen des Siliciums mit Benzol und mit Biphenyl. *J. Organomet. Chem.* **1992**, *434*, 35–44.

S3 Gilman, H.; Smith, C. L. Tetrakis(trimethylsilyl)silane. *J. Organomet. Chem.* **1967**, *8*, 245–253.

S4 Yamaguchi, S.; Jin, R.-Z.; Tamao, K.; Shiro, M. Synthesis of a Series of 1,1-Difunctionalized Siloles. *Organometallics* **1997**, *16*, 2230–2232.

S5 Pöcheim, A.; Özpınar, G. A.; Müller, T.; Baumgartner, J.; Marschner, C. The Combination of Cross-Hyperconjugation and σ-Conjugation in 2,5-Oligosilanyl Substituted Siloles. *Chem. Eur. J.* **2020**, *26*, 17252–17260.

S6 Morris, G. A.; Freeman, R. Enhancement of Nuclear Magnetic Resonance Signals by Polarization Transfer. *J. Am. Chem. Soc.* **1979**, *101*, 760–762.

S7 Helmer, B. J.; West, R. Enhancement of ^{29}Si NMR Signals by Proton Polarization Transfer. *Organometallics* **1982**, *1*, 877–879.

S8 Blinka, T. A.; Helmer, B. J.; West, R. Polarization Transfer NMR Spectroscopy for Silicon-29: The INEPT and DEPT Techniques. *Adv. Organomet. Chem.* **1984**, *23*, 193–218.

S9 SAINTPLUS: Software Reference Manual, Version 6.45, Bruker-AXS, Madison, WI, 1997-2003.

S10 Blessing, R. H. An Empirical Correction for Absorption Anisotropy. *Acta Cryst. A* **1995**, *51*, 33–38.

S11 Sheldrick, G. M. (2003). SADABS. Version 2.10. Bruker AXS Inc., Madison, USA. *Sheldrick, G. M. (2003). SADABS. Version 2.10. Bruker AXS Inc., Madison, USA.*

S12. Dolomanov, O. V.; Bourhis, L. J.; Gildea, R. J.; Howard, J. A. K.; Puschmann, H. OLEX2: A Complete Structure Solution, Refinement and Analysis Program. *J. Appl. Cryst.* **2009**, *42*, 339–341.

S13 Sheldrick, G. M. Crystal Structure Refinement with SHELXL. *Acta Cryst. C* **2015**, *71*, 3–8.

S14 Farrugia, L. J. *WinGX* and *ORTEP* for *Windows*: An Update. *J. Appl. Cryst.* **2012**, *45*, 849–854.

S15 POVRAY 3.6. Persistence of Vision Pty. Ltd.: Williamstown, Victoria, Australia, 2004. Available online: http://www.povray.org/download/ (accessed on 09.07.2008).

S16 Gaussian 16, Revision C.01, M. J. Frisch, G. W. Trucks, H. B. Schlegel, G. E. Scuseria, M. A. Robb, J. R. Cheeseman, G. Scalmani, V. Barone, G. A. Petersson, H. Nakatsuji, X. Li, M. Caricato, A. V. Marenich, J. Bloino, B. G. Janesko, R. Gomperts, B. Mennucci, H. P. Hratchian, J. V. Ortiz, A. F. Izmaylov, J. L. Sonnenberg, D. Williams-Young, F. Ding, F. Lipparini, F. Egidi, J. Goings, B. Peng, A. Petrone, T. Henderson, D. Ranasinghe, V. G. Zakrzewski, J. Gao, N. Rega, G. Zheng, W. Liang, M. Hada, M. Ehara, K. Toyota, R. Fukuda, J. Hasegawa, M. Ishida, T. Nakajima, Y. Honda, O. Kitao, H. Nakai, T. Vreven, K. Throssell, J. A. Montgomery, Jr., J. E. Peralta, F. Ogliaro, M. J. Bearpark, J. J. Heyd, E. N. Brothers, K. N. Kudin, V. N. Staroverov, T. A. Keith, R. Kobayashi, J. Normand, K. Raghavachari, A. P. Rendell, J. C. Burant, S. S. Iyengar, J. Tomasi, M. Cossi, J. M. Millam, M. Klene, C. Adamo, R. Cammi, J. W. Ochterski, R. L. Martin, K. Morokuma, O. Farkas, J. B. Foresman, and D. J. Fox, Gaussian, Inc., Wallingford CT, 2016.

S17 Y. Zhao, N. E. Schultz and D. G. Truhlar, *J. Chem. Theory Comput.* **2006**, *2*, 364.

S18 a) B. P. Pritchard, D. Altarawy, B. Didier, T.D.Gibsom, T. L. Windus, *J. Chem. Inf. Model.* **2019**, *59*, 4814-4820. b) D. Feller, *J. Comput. Chem*. **1996**, *17*, 1571-1586. c) K. L. Schuchardt, B. T. Didier, T. Elsethagen, L. Sun, V. Gurumoorthi, J. Chase, J. Li, Jun, T. L. Windus, *J. Chem. Inf. Model*. **2007**, *47*, 1045-1052.

S19 F. Weigend, R. Ahlrichs *Phys. Chem. Chem. Phys.* **2005**, *7*, 3297.

S20 K. Fukui, *Acc. Chem. Res.* **1981**, *14*, 363.
